# Brg1 modulates enhancer activation in mesoderm lineage commitment

**DOI:** 10.1242/dev.109496

**Published:** 2015-04-15

**Authors:** Jeffrey M. Alexander, Swetansu K. Hota, Daniel He, Sean Thomas, Lena Ho, Len A. Pennacchio, Benoit G. Bruneau

**Affiliations:** 1 Gladstone Institute of Cardiovascular Disease, San Francisco, CA 94158, USA; 2 Roddenberry Center for Stem Cell Biology and Medicine at Gladstone, San Francisco, CA 94158, USA; 3 Institute of Medical Biology, A*STAR, Singapore138648; 4 Genomics Division, Lawrence Berkeley National Laboratory, Berkeley, CA 94720, USA; 5 United States Department of Energy, Joint Genome Institute, Walnut Creek, CA 94598, USA; 6 Department of Pediatrics, University of California, San Francisco, CA 94143, USA; 7 Cardiovascular Research Institute, University of California, San Francisco, CA 94158, USA

**Keywords:** Chromatin, Enhancers, Gene expression, Histone modification, Mesoderm, Stem cells

## Abstract

The interplay between different levels of gene regulation in modulating developmental transcriptional programs, such as histone modifications and chromatin remodeling, is not well understood. Here, we show that the chromatin remodeling factor *Brg1* is required for enhancer activation in mesoderm induction. In an embryonic stem cell-based directed differentiation assay, the absence of Brg1 results in a failure of cardiomyocyte differentiation and broad deregulation of lineage-specific gene expression during mesoderm induction. We find that Brg1 co-localizes with H3K27ac at distal enhancers and is required for robust H3K27 acetylation at distal enhancers that are activated during mesoderm induction. *Brg1* is also required to maintain Polycomb-mediated repression of non-mesodermal developmental regulators, suggesting cooperativity between Brg1 and Polycomb complexes. Thus, *Brg1* is essential for modulating active and repressive chromatin states during mesoderm lineage commitment, in particular the activation of developmentally important enhancers. These findings demonstrate interplay between chromatin remodeling complexes and histone modifications that, together, ensure robust and broad gene regulation during crucial lineage commitment decisions.

## Abstract (precis)

**SUMMARY**: The chromatin remodeling factor Brg1 is essential for mesoderm induction and, by modulating active and repressive chromatin states, is involved in promoting the activation of dynamic enhancers.

## INTRODUCTION

The emergence of individual cell types during development relies on the correct sets of genes becoming activated, while inappropriate sets of genes are simultaneously repressed. This process is achieved in large part by modifying chromatin structure, which packages the genome within the nucleus and affects multiple facets of gene regulation (Ho and Crabtree, 2010).

Histone modifications annotate the genome by marking active, repressed and other functional domains singly or in combination (Zhou et al., 2011). For example, trimethylation of histone H3 lysine 27 (H3K27me3), deposited by the Polycomb repressive complex 2 (PRC2), is associated with gene silencing (Surface et al., 2010). In embryonic stem cells (ESCs), PRC2 targets a broad group of developmental regulators for silencing to ensure appropriate lineage-specific gene expression (Surface et al., 2010). Conversely, acetylated histone H3 lysine 27 (H3K27ac) is a hallmark of active chromatin and, in particular, of enhancers, non-coding DNA elements that regulate tissue-specific gene expression patterns (Calo and Wysocka, 2013). Enhancer occupancy by H3K27ac is highly dynamic during cellular differentiation, yet the factors that modulate H3K27ac occupancy at promoters and enhancers remain poorly understood.

Eukaryotes use ATP-dependent chromatin remodelers to unwind, slide and/or evict individual nucleosomes (Ho and Crabtree, 2010; Wu et al., 2009). In mammals, the Swi/Snf-like Brg1/Brm-associated factor (BAF) chromatin-remodeling complexes include 10-12 interchangeable subunits and function in regulating cell-cycle progression, DNA repair and development (Ho and Crabtree, 2010). Remodeling activity is mediated by the ATPase subunit, which is encoded by either *Brg1* (also known as *Smarca4*) or the related gene *Brm* (also known as *Smarca2*). *Brg1* is essential for embryonic development and maintenance of pluripotency, whereas *Brm* knockout mice are viable (Bultman et al., 2000; Ho et al., 2009; Reyes et al., 1998), arguing that *Brg1* is the consequential ATPase during development. *Brg1* is required in numerous tissue and cell types *in vivo* (Ho and Crabtree, 2010), including multiple cell types within the cardiac lineage (Hang et al., 2010; Stankunas et al., 2008; Takeuchi and Bruneau, 2009; Takeuchi et al., 2011). How BAF complexes function to regulate gene expression within these cell lineages is still poorly understood.

A comprehensive picture of *Brg1* function during development necessitates detailed understanding not only of the regulatory loci bound by Brg1 but also its functional activity at these regions, including how this activity facilitates the transitions between distinct chromatin states – marked by histone modifications – that occur during cell differentiation. Such questions bear on fundamental features of chromatin regulation, namely how epigenetic modification and chromatin remodeling are deployed during development to modify the chromatin template in a coordinated fashion. Brg1 is found at distal regulatory regions (Euskirchen et al., 2011; Hu et al., 2011; Morris et al., 2014; Rada-Iglesias et al., 2011; Yu et al., 2013), but the role of Brg1 at these regions remains poorly understood. Here, we investigate the function of *Brg1* during embryonic stem cell differentiation. We find that loss of *Brg1* leads to disruption of cardiomyocyte differentiation and dysregulation of lineage-specific gene expression during mesoderm induction. Furthermore, we find that *Brg1* is required for robust H3K27 acetylation, predominately at distal enhancers that transition from inactive to active states during mesoderm induction. *Brg1* is also required to maintain Polycomb-mediated repression of non-mesodermal developmental regulators through deposition of H3K27me3, suggesting cooperativity between Brg1 and Polycomb complexes.

## RESULTS

### Essential BAF complex subunits are enriched at early stages of cardiac differentiation

**Fig. 1.**
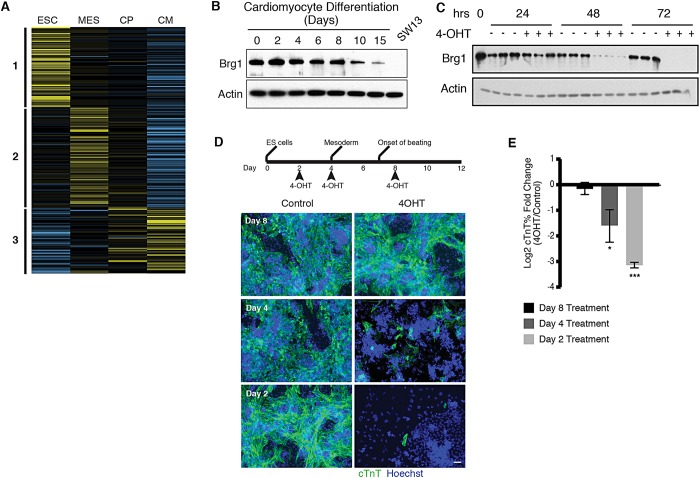
***Brg1* is required for directed differentiation of ESCs to cardiomyocytes (CMs).** (A) Heat map representation of clustering analysis of RNA expression of chromatin regulators at four stages of directed CM differentiation identifies three expression patterns. Chromatin regulators include genes annotated with GO terms GO0006338 and GO00016569 and additional known regulators. (B) Western blot analysis demonstrates reduced abundance of Brg1 at late stages of CM differentiation. Lysate from the adrenal carcinoma SW13, which does not express Brg1, was used as a negative control. Actin was used as a loading control. (C) Western blot analysis of a 4-OHT treatment time course in *Brg1^f/f^; Actin-CreER* ESCs. Loss of Brg1 expression is near complete after 48 h of 4-OHT treatment. (D) Control (vehicle only, THF) or 4-OHT was added after 2, 4 and 8 days of differentiation to mediate *Brg1* deletion, and the presence of cardiomyocytes was determined by immunofluorescence for cTnT at day 12. Scale bar: 50 µm. (E) Comparison of percentage of cTnT^+^ cells for control or 4-OHT-treated cultures measured by flow cytometry. *P<0.05, ***P<0.001; one-sample *t*-test.

To gain deeper insight into how chromatin is regulated during cardiac differentiation, we analyzed our published expression datasets (Wamstad et al., 2012) to identify the expression patterns of known chromatin regulators. We selected genes annotated with involvement in chromatin remodeling (Gene ontology category GO0006338) or covalent chromatin modification (GO00016569), and clustered their gene expression patterns across four stages of cardiomyocyte differentiation: ESCs, mesodermal precursors (MES), cardiac precursors (CP) or functional cardiomyocytes (CMs) (supplementary material Table S1). This analysis classified chromatin regulators into three expression patterns (Fig. 1A). We identified one cluster with high expression in ESCs and reduced expression upon exit of the pluripotent state. Within this group were the *de novo* DNA methyltransferase *Dnmt3b*, which modulates DNA methylation levels in the early mouse embryo (Okano et al., 1999), and *Dpy30*, a member of MLL family complexes that is required for differentiation of ESCs (Jiang et al., 2011) (Table 1). Expression in a second cluster peaked at the MES stage, followed by downregulation in differentiated CMs. Many BAF complex subunits demonstrated this expression pattern, including the essential core subunits *Baf57* (*Smarce1* – Mouse Genome Informatics) and *Baf47* (*Smarcb1* – Mouse Genome Informatics) and the enzymatic subunit *Brg1* (Ho and Crabtree, 2010; Wu et al., 2009). The last expression cluster demonstrated increased CM expression; within this group were *Smyd1*, a known regulator of cardiac development (Gottlieb et al., 2002); *Hdac9*, which modulates the hypertrophic response (Zhang et al., 2002); and *Jmjd3* (*Kdm6b* – Mouse Genome Informatics), a histone H3 lysine 27 demethylase. This cluster also contained multiple components of BAF complexes, including *Baf60c* (*Smarcd3* – Mouse Genome Informatics), *Brm*, *Baf170* (*Smarcc2* – Mouse Genome Informatics), *Baf45c* (*Dpf3* – Mouse Genome Informatics), *Baf45d* and *Baf250b* (*Arid1b* – Mouse Genome Informatics), suggesting that BAF complexes undergo subunit switching during cardiomyocyte differentiation analogous to that observed in the nervous system (Ho and Crabtree, 2010; Wu et al., 2009).

**Table 1.**
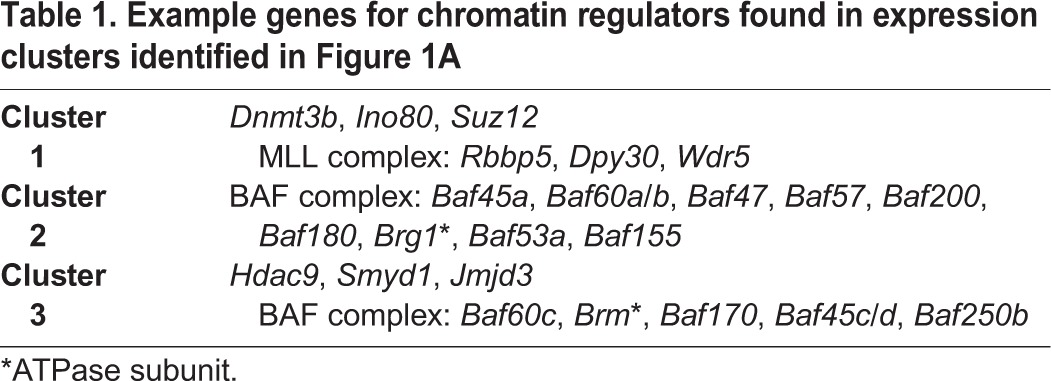
**Example genes for chromatin regulators found in expression clusters identified in Figure 1A**

### *Brg1* is required to induce mesoderm and cardiac markers from embryonic stem cells

Dynamic expression of BAF complex subunits suggested that these complexes play distinct roles at different stages of cardiac differentiation. We confirmed that Brg1 protein is more abundant at early stages of cardiac differentiation than in late-stage cultures enriched for differentiated CMs (Fig. 1B). The enrichment of *Brg1* in mesoderm precursors suggested that BAF complexes perform a broad and uncharacterized function in the early progenitors of the cardiac lineage.

To investigate the function of *Brg1* at distinct stages of cardiac differentiation, we used directed CM differentiation (see supplementary material Methods) in a mouse ESC line with two floxed alleles of *Brg1* and a constitutively expressed Cre recombinase-estrogen receptor fusion protein (*Brg1*^f/f^;*Actin-CreER*) (Ho et al., 2011, 2009). Adding 4-hydroxytamoxifen (4-OHT) led to efficient deletion of the floxed allele and loss of Brg1 protein, which was near-complete 48 h after 4-OHT treatment and undetectable by 72 h (Fig. 1C). Treatment of differentiating cultures with 4-OHT allowed for controlled deletion of *Brg1* at specific stages of differentiation and allowed for the comparison of control and treatment groups within a single differentiation, which limited confounding effects from differentiation variability. We added 4-OHT at three time points: during the induction of MES (day 2), as mesodermal precursors are differentiating towards CMs (day 4), and after the appearance of beating CMs (day 8) (Fig. 1D). Cultures treated with 4-OHT at day 8 showed no discernable differences from control cultures: they continued to contract many days after addition of 4-OHT and had comparable numbers of cardiac troponin T (cTnT; Tnnt2 – Mouse Genome Informatics)-positive CMs (Fig. 1D,E). By contrast, cultures treated with 4-OHT at day 2 or day 4 had fewer cTnT^+^ CMs than controls. This was most striking in cultures treated with 4-OHT at day 2, which had a near-complete loss of cTnT^+^ CMs. Whereas control-treated cultures expand to generate dense layers, containing multiple fibers of interconnected CMs after mesoderm induction, cultures treated with 4-OHT at day 2 failed to expand in these conditions, giving rise to a sparse monolayer of differentiated cells (supplementary material Fig. S1A,B). Treatment of wild-type ESCs undergoing the same differentiation protocol with 4-OHT did not affect their ability to differentiate (supplementary material Fig. S1C). Thus, *Brg1* is required for the differentiation of embryonic stem cells to CMs.

**Fig. 2.**
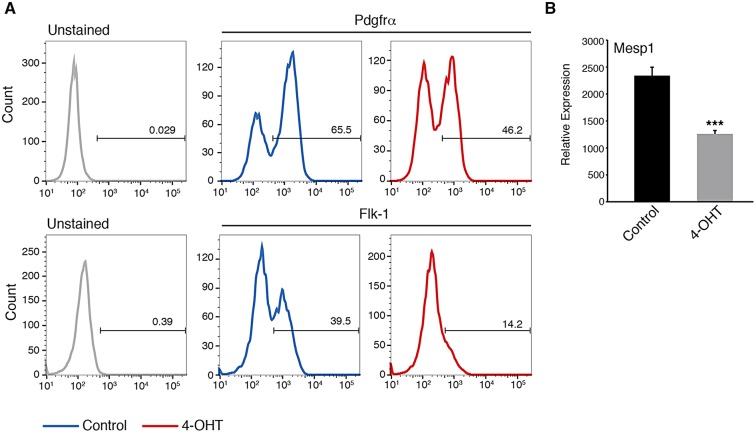
**Brg1 is required for robust induction of mesodermal markers.** (A) Flow cytometry for Pdgfra and Flk-1 at day 4 of differentiation demonstrates a lower percentage of cells expressing these mesoderm markers in cultures deleted for *Brg1*. (B) Quantitative PCR demonstrates reduced expression of *Mesp1* in day 4 cultures depleted for *Brg1*. ***P<0.001; Student's *t*-test.

As addition of 4-OHT at day 2 would lead to deletion of *Brg1* during mesoderm induction, we examined mesodermal markers during differentiation of ESCs. We differentiated *Brg1*^f/f^;*Actin-CreER* ESCs for 2 days as embryoid bodies (EBs) in serum-free medium and induced MES by treating these cultures with Vegf, activin A (Inhba – Mouse Genome Informatics) and Bmp4 in the presence of 4-OHT or vehicle control for 40 h, analogous to the first 4 days of our directed CM differentiation protocol. We measured the induction of Flk-1 (also known as *Kdr*) and Pdgfra, receptor tyrosine kinases expressed on cardiogenic mesodermal cells (Kattman et al., 2011). Whereas control cultures showed robust induction of Pdgfra by flow cytometry, *Brg1*-deleted cultures showed a clear reduction in the number of Pdgfra- and Flk1-expressing cells (*n*=3; Fig. 2A). Similarly, *Brg1*-deleted cultures showed reduced expression of the mesodermal marker *Mesp1* (Fig. 2B). The remaining expression still observed for Pdgfra, Flk-1 and *Mesp1* in these experiments might be the result of residual Brg1 activity, as mesoderm is induced concomitant with addition of 4-OHT, leading to a gradual loss of Brg1 protein during mesoderm induction. Taken together, these data demonstrate that *Brg1* is required in ESCs for robust induction of molecular markers of mesoderm.

### *Brg1* is required for gene activation and maintenance of gene repression during mesoderm differentiation

**Fig. 3.**
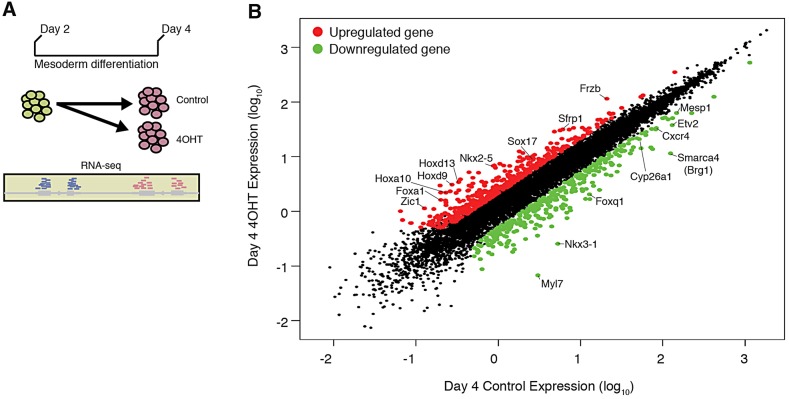
**Global expression analysis of *Brg1*-deleted mesodermal cultures reveals dysregulation of essential developmental genes.** (A) Cartoon of the RNA-seq experimental design. (B) Day 4 expression in control samples plotted versus day 4 expression in 4-OHT-treated samples. Genes significantly changed (>twofold change, FDR=1%) are colored in red and green for upregulated and downregulated, respectively. Example genes are highlighted.

To identify the *Brg1*-dependent transcriptional program during mesoderm differentiation, we collected cultures before mesoderm induction (day 2) and cultures 40 h after treatment with Vegf, activin A and Bmp4 (day 4) that had been treated with either 4-OHT or control, and measured global gene expression by RNA-seq (Fig. 3A). This allowed identification of the transcriptional changes that occur normally during mesoderm differentiation, in addition to genes differentially expressed between 4-OHT and control. In this way, we could determine whether *Brg1*-dependent genes demonstrate a common expression pattern during mesoderm induction. Using stringent criteria (FDR=1%, fold change ≥2), we identified 350 downregulated and 502 upregulated genes in *Brg1*-deleted cultures (Fig. 3B; supplementary material Table S2). This analysis demonstrated that *Brg1* was downregulated more than tenfold in *Brg1*-deleted cultures, confirming the efficacy of the genetic deletion. Among the downregulated genes were *Flk1* and *Mesp1*, mesodermal markers that demonstrated reduced induction by flow cytometry or quantitative PCR. We also found other genes essential for mesoderm development, of which the expression was reduced by loss of *Brg1*, including *Cxcr4*, *Cyp26a1* and *Snai1*. *Cxcr4* is expressed in Flk-1/Pdgfra-expressing mesoderm and mediates differentiation towards the cardiac lineage. *Cyp26a1* modulates retinoic acid signaling, a crucial regulator of mesodermal patterning (Aulehla and Pourquié, 2010; Chiriac et al., 2010; Nelson et al., 2008). *Snai1* is a transcriptional repressor that controls epithelial-to-mesenchymal transition (EMT) and mesoderm morphology *in vivo* (Carver et al., 2001). Gene ontology (GO) analysis revealed that downregulated genes were enriched for genes involved in cell adhesion as well as those associated broadly with development (multicellular organismal process) and signaling (molecular transducer activity) (Table 2; supplementary material Table S3). These findings are consistent with defective induction of mesodermal genes in *Brg1*-deficient cultures. *Pdgfra*, which had reduced expression, as measured by flow cytometry (Fig. 2A), was downregulated 1.93-fold in our RNA-seq analysis and thus fell slightly below our stringent criteria for significance. Therefore, our statistical cutoff is probably a conservative estimate of *Brg1*-dependent gene expression.

**Table 2.**
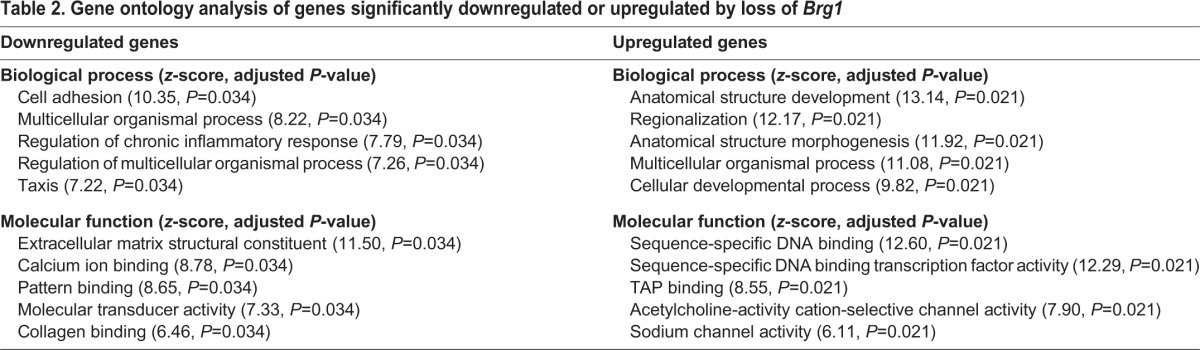
**Gene ontology analysis of genes significantly downregulated or upregulated by loss of *Brg1***

Examination of the genes upregulated by loss of *Brg1* (Fig. 3B) revealed Wnt signaling ligands (*Wnt8a*, *Wnt9a*, *Wnt7b*, *Wnt4*, *Wnt10a* and *Wnt6*), Wnt antagonists (*Sfrp1* and *Frzb*), and numerous developmental transcription factors (TFs). The latter included TFs from the Fox, Tbx, Mef2, Dlx, Runx, Pax, Lhx, Six, Nkx, Sox, Pou, Cdx, Irx and Hox TF families. GO analysis was consistent with this finding; upregulated genes demonstrated strong enrichment for genes associated with development, morphogenesis and transcription factor function (Table 2). Investigation of expression data from a broad range of murine tissues and cell types confirmed that upregulated TFs are expressed in many distinct and non-overlapping lineages (supplementary material Fig. S2), demonstrating that loss of *Brg1* does not result in differentiation of ESCs towards a single, non-mesodermal lineage. Strikingly, many (18 of 38) *Hox* genes, representing all four *Hox* clusters, were upregulated in *Brg1*-deleted mesoderm.

Within genes significantly upregulated by loss of *Brg1* were numerous TFs that function during heart development, despite the striking defect for these cultures to generate cardiomyocytes at subsequent stages. This group included the well-characterized regulators of skeletal and cardiac myogenesis *Myocd* and *Mef2b* as well as conserved cardiogenic factors *Tbx5* and *Nkx2-5.* Of particular note was *Nkx2-5*, the expression of which increased more than sevenfold in *Brg1*-deficient mesoderm. Our previous studies (Wamstad et al., 2012) have shown that these factors are expressed at low levels in mesodermal cultures, becoming robustly expressed only later, concomitant with the onset of cardiomyocyte differentiation. We did not observe broad upregulation of markers of cardiomyocytes in these cultures, suggesting instead that the upregulation of these cardiogenic TFs reflects the broader misexpression of inappropriate developmental regulators in *Brg1*-deficient mesodermal cultures.

**Fig. 4.**
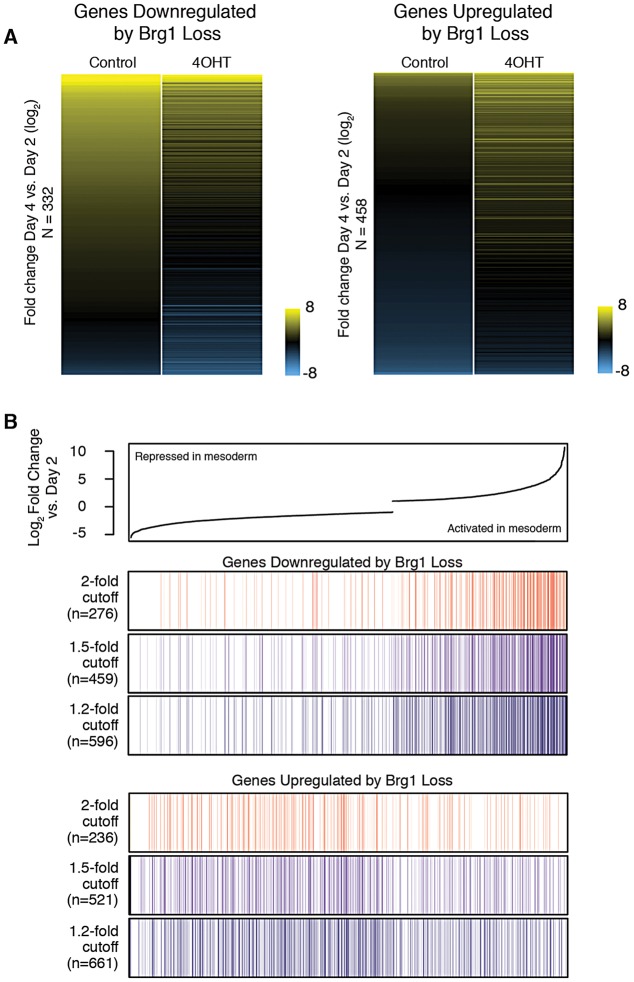
***Brg1* is required for gene activation and maintenance of gene silencing during mesoderm differentiation.** (A) Heat map of log_2_-fold change in expression during mesoderm differentiation for genes significantly downregulated or upregulated by loss of *Brg1*. (B) (Top) Genes are rank-ordered based on log_2_-fold change in expression between day 4 control and day 2 (normal mesoderm differentiation). Only genes significantly changed during mesoderm differentiation are shown. (Bottom) Color bars depict distribution of genes downregulated by loss of *Brg1* by numerous fold change cut-offs. Colors vary only for ease of visualization and do not correlate to numerical values. Each vertical line represents a single dysregulated gene. Genes downregulated by loss of *Brg1* cluster to the right, suggesting a role for *Brg1* in gene activation during mesoderm differentiation. Conversely, genes upregulated by loss of *Brg1* are more often found repressed during mesoderm differentiation. *N* indicates the number of *Brg1*-dependent genes shown.

*Brg1* might function to facilitate dynamic changes in gene expression that occur during mesoderm induction or might be required to maintain active and repressed transcriptional states. To better understand the function of *Brg1* in transcriptional regulation of mesoderm differentiation, we investigated the expression patterns of *Brg1*-dependent genes during this process. We rank-ordered genes based on fold change in gene expression during normal mesoderm differentiation (day 4 control versus day 2) and compared normal and *Brg1*-deleted mesoderm differentiation. We limited our analysis to only those genes measured by RNA-seq in all three experimental conditions. We found that the majority (82%) of genes downregulated by loss of *Brg1* are activated during normal mesoderm induction (Fig. 4A, left bar); *Brg1* deletion during mesoderm induction led to less robust activation for these genes (Fig. 4A, right bar). By contrast, genes upregulated by loss of *Brg1* showed a tendency for repression during normal mesoderm differentiation. We found that upregulated genes were generally expressed at very low levels in normal mesodermal cultures (supplementary material Fig. S3). Loss of *Brg1* leads to derepression of these genes during mesoderm differentiation. Collectively, our global gene expression analysis supports broad roles for *Brg1* in gene activation and maintaining gene repression of key developmental regulators during mesoderm differentiation of ESCs.

We next investigated to what extent *Brg1* is required for transcriptional change during mesoderm induction. We identified genes differentially expressed between day 4 control and day 2 (FDR=1%, fold change ≥2), categorized these genes as either activated or repressed during mesoderm induction, and overlapped these gene sets with *Brg1*-dependent genes. This analysis revealed that 18% of genes activated during normal mesoderm differentiation (i.e. significantly higher expression at day 4) had reduced expression in *Brg1*-deleted mesodermal cultures, compared with just 2% of repressed genes. Mesoderm-activated genes were considerably enriched for those dependent on *Brg1* for expression (Fig. 4B). This demonstrates that a substantial proportion of the mesoderm transcriptional program requires *Brg1* for proper activation.

### *Brg1* is required for H3K27ac enrichment at dynamically activated enhancers proximal to dysregulated genes

To better understand the mechanism by which *Brg1* affects the mesodermal transcriptional program, we defined the genomic regions bound by Brg1 in mesodermal cultures. To this end, we used ESCs harboring a *Brg1* allele that encodes a 3×-FLAG epitope tag fused to the C-terminal end of Brg1 targeted to the endogenous *Brg1* locus (Attanasio et al., 2014). We confirmed the expression of Brg1-FLAG in cultured pluripotent ESCs (supplementary material Fig. S4). Purification of Brg1-FLAG using an anti-Flag column yielded a staining pattern that closely resembled those published for BAF complexes (Ho et al., 2009; Wang et al., 1996) (supplementary material Fig. S4), consistent with Brg1-FLAG incorporation into BAF complexes. Mass spectrometry of isolated complexes revealed a composition highly similar to previously reported esBAF complexes (Ho et al., 2009) (data not shown). Mice homozygous for the Brg1-FLAG allele are viable, further indicating that the allele is fully functional, and ChIP-seq data, obtained in mouse tissues using this Brg1-FLAG allele, strongly correlated with published Brg1 ChIP-seq data obtained with antisera (Attanasio et al., 2014). We differentiated Brg1-FLAG ESCs to mesodermal precursors and performed chromatin immunoprecipitation and deep sequencing (ChIP-seq) on biological duplicate samples. FLAG ChIP-seq replicates overlapping H3K27ac-enriched regions were well correlated (r^2^=0.71) and showed modest enrichment that probably reflects the transient and dynamic nature of chromatin remodeler binding. To identify high-confidence Brg1-bound regions from these data, we overlapped statistically enriched peaks identified through an input-corrected Poissonian model across both replicates (Marson et al., 2008) and identified 3027 bound regions distributed throughout the mouse genome (see supplementary material Methods, Table S4). Given the modest enrichment of our FLAG ChIP-seq dataset, we expect these regions, statistically enriched in both biological replicates, to represent a conservative estimate of Brg1 occupancy.

**Fig. 5.**
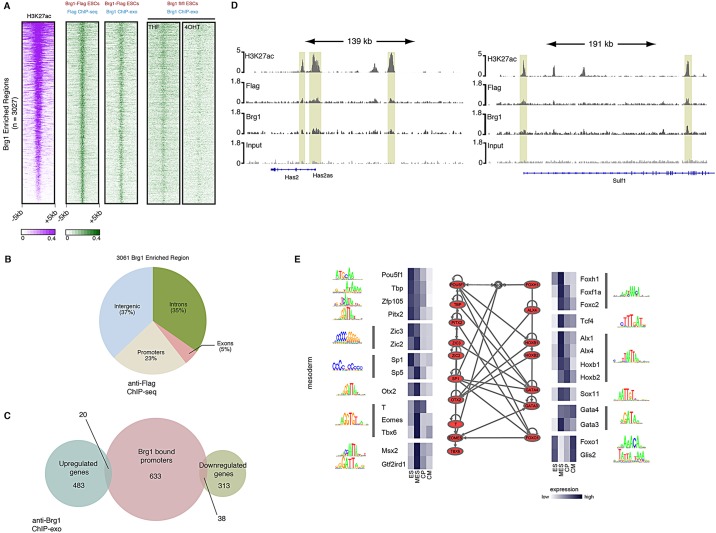
***Brg1* co-localizes with H3K27ac genome-wide.** (A) Density of ChIP-seq reads for H3K27ac, anti-FLAG ChIP-seq and anti-Brg1 ChIP-exo in Brg1-FLAG ESCs, and anti-Brg1 ChIP-exo in THF- and 4-OHT-treated *Brg1^fl/fl^* ESCs. Plots show ±5 kb around the midpoint of each Brg1-enriched region ranked according to H3K27ac density, and demonstrate significant co-localization of Brg1 with H3K27ac. Brg1 is detected at these regions across distinct cell lines and ChIP antibodies, and is lost upon genetic deletion. (B) Distribution of Brg1-enriched regions across the mouse genome. (C) Overlap between genes with Brg1 enrichment within 2.5 kb of the TSS and genes dysregulated by loss of *Brg1* demonstrates little overlap between *Brg1*-dependent genes and Brg1-bound promoters. (D) Co-localization of Brg1 and H3K27ac at example genomic regions. *y*-axis shows reads per bin per million. (E) Motifs enriched at Brg1-occupied enhancer elements. TRANSFAC positional Weight Matrices for each significantly (q<0.001) enriched motif is shown next to transcription factors known to bind these motifs that are expressed at the mesoderm stage (interquartile range-normalized RPKM values are shown). Known regulatory interactions, as identified using the Ingenuity Pathway analysis, are also annotated for each gene.

To validate Brg1-FLAG-bound regions, we performed ChIP-exo, using an antibody against endogenous Brg1 (Morris et al., 2014) in Brg1-FLAG ESCs differentiated to mesodermal precursors in biological duplicates. As expected, our 3027 Brg1-FLAG-bound regions demonstrated modest but clear enrichment for Brg1 in the ChIP-exo dataset (Fig. 5A), with occupancy characteristics similar to published results (Morris et al., 2014; Shi et al., 2013). Correlation between anti-FLAG and anti-Brg1 ChIP over H3K27ac-enriched regions is 0.52. To further validate these regions and the specificity of the antibody, we performed Brg1 ChIP-exo on differentiating *Brg1*^f/f^;*Actin-CreER* ESCs treated with either THF (control) or 4-OHT (deletion of *Brg1*) for 48 h. Brg1 enrichment, modest but clear in THF-treated samples, was greatly reduced in *Brg1*^f/f^;*Actin-CreER* ESCs treated with 4-OHT. Taken together, we have defined Brg1-bound regions consistently enriched by different methods, which are sensitive to genetic deletion of Brg1. We acknowledge that the modest enrichment only allows the identification of high-confidence high-enrichment regions; therefore, our conclusions regarding the direct function of Brg1 are limited to these regions.

Comparison of Brg1-bound regions with genomic annotations revealed Brg1 binding within gene promoters, introns, exons and intergenic regions (Fig. 5B). We identified 691 genes with reproducible binding of Brg1 within 2.5 kb of the transcriptional start site. Some genes in this group were also significantly changed in our RNA-seq dataset. However, an intersection of Brg1-bound promoters with *Brg1*-dependent genes revealed little overlap (Fig. 5C), as observed for other chromatin remodelers (Gelbart et al., 2005; Sala et al., 2011). We find the majority of genes with Brg1 promoter enrichment do not show significant changes in gene expression. Moreover, most *Brg1*-dependent genes lacked robust Brg1 binding within the promoter. This suggests that Brg1 does not predominately modulate gene expression through promoter regulation in differentiating mesoderm.

Brg1 localizes to well-characterized enhancers (Bultman et al., 2005) and predicted enhancers genome-wide (Euskirchen et al., 2011; Hu et al., 2011; Rada-Iglesias et al., 2011; Yu et al., 2013). Given that only 23% of Brg1 peaks were found within promoter regions, we hypothesized that Brg1 functions at distal enhancers. To test this, we generated genome-wide maps of H3K27ac in control and 4-OHT-treated mesodermal cultures in biological duplicates. H3K27ac marks active enhancers and can be used to identify putative distal regulatory elements genome-wide (Calo and Wysocka, 2013). Comparison of Brg1-FLAG and H3K27ac ChIP-seq signals at Brg1-enriched loci showed substantial enrichment for H3K27ac at Brg1-bound regions (Fig. 5A; see also Attanasio et al., 2014). Moreover, our ChIP-seq data revealed a high correlation between Brg1 and H3K27ac signals throughout the genome (Fig. 5D; supplementary material Fig. S5). Using our genome-wide maps of H3K27ac, we identified 16,724 putative enhancer regions distal (>2.5 kb) from the transcriptional start site. Strikingly, we found that 68% of Brg1-bound regions distal to transcriptional start sites overlapped with predicted enhancer regions (*P*<0.0001; 10,000 permutations). Thus, Brg1 associates with a proportion of H3K27ac-marked enhancers genome-wide in mesodermal cultures.

We searched Brg1-bound enhancers for enriched transcription factor DNA binding motifs that might predict a mechanism for site-specific recruitment of Brg1. H3K27ac^+^ Brg1-bound regions were scanned using the ‘match’ algorithm of TRANSFAC. A number of motifs were significantly enriched (q<0.001), with many belonging to well-known regulators of mesodermal differentiation, including T-box, GATA and Fox factors, which function in a highly interactive network (Fig. 5E). Although enrichments were highly significant, fold enrichment in motif abundance between Brg1-associated enhancers and all enhancers was modest (between 1.11- and 1.95-fold enrichment). We conclude that Brg1-bound enhancers are enriched for specific developmental TFs, but it is unlikely that these factors alone direct Brg1 occupancy, and might instead reflect bias in Brg1 recruitment to developmentally regulated enhancers.

**Fig. 6.**
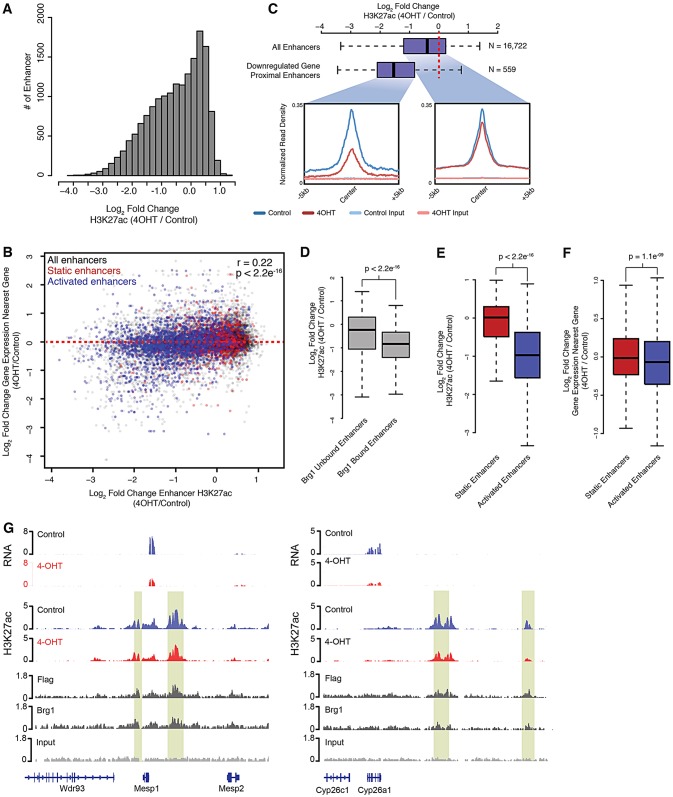
***Brg1* is required for enhancer activation in differentiating mesodermal cultures.** (A) Histogram of log_2_-fold change in H3K27ac at predicted enhancers. (B) Scatterplot of log_2_-fold change of H3K27ac at predicted enhancers and the log_2_-fold change in gene expression between day 4 4-OHT and day 4 control cultures of the nearest gene to each enhancer plotted. Red and blue dots highlight enhancers marked by H3K27ac in undifferentiated ESCs and mesodermal cultures (static enhancers), and those marked in mesoderm cultures only (activated enhancers), respectively. (C) Box plots of log_2_-fold change of subsets of predicted enhancers with read density profiles of each enhancer cohort. Enhancers associated with downregulated genes include enhancers of which the most proximal gene is significantly downregulated in *Brg1*-deleted mesoderm. Downregulated gene-associated enhancers show greater average loss in H3K27ac than all enhancers. (D) Box plots of log_2_-fold change in H3K27ac for predicted enhancers in *Brg1*-deficient cultures. Enhancers are separated into Brg1-bound and unbound cohorts based on the presence or absence of a Brg1-enriched region, respectively. (E,F) Box plots of log_2_-fold change in H3K27ac (E) or expression of the nearest gene (F) for static and activated enhancers in *Brg1*-deficient cultures. (G) H3K27ac at putative enhancer regions proximal to the *Mesp1* and *Cyp26a1* genes. *y*-axis shows reads per bin per million.

The presence of Brg1 at enhancers suggests that Brg1 regulates transcriptional activation during mesoderm induction through modulation of enhancer activity. We therefore asked whether H3K27ac levels were altered in *Brg1*-deleted mesodermal cultures. To this end, we compared H3K27ac genome-wide maps from control and 4-OHT-treated mesodermal cultures, and rank-ordered promoter and enhancer regions based on fold change in H3K27ac in *Brg1*-deleted cultures. We found that levels of H3K27ac were largely unchanged at promoter regions (median log_2_-fold change=0.07), although downregulated genes showed clear reductions in H3K27ac levels proximal to the TSS, probably reflecting decreased transcriptional activity at these genes (supplementary material Fig. S6). In contrast to promoter regions, we observed a global reduction in H3K27ac levels at predicted enhancer regions in *Brg1*-deleted cultures (median log_2_-fold change=−0.39) (Fig. 6A). Consistent with a functional role for enhancer activity in the transcriptional changes seen in *Brg1*-deleted cultures, we observed a correlation between changes in H3K27ac seen at an enhancer and changes in expression of its nearest gene (Fig. 6B). This relationship was observed despite limitations of computational approaches in predicting enhancer-gene regulatory pairs. Furthermore, we found that enhancers proximal to significantly downregulated genes showed greater reductions in H3K27ac compared with all putative enhancers (Fig. 6C). These findings support a functional role for *Brg1*-dependent enhancer activity in the transcriptional control of mesoderm differentiation.

To investigate whether Brg1 regulates enhancer activity directly through its recruitment to these loci, we partitioned mesodermal enhancers into Brg1-bound or Brg1-unbound cohorts. We found that Brg1-bound enhancers showed greater losses in H3K27ac than those without Brg1-enrichment, providing evidence that Brg1 directly modulates H3K27ac at enhancers (Fig. 6D). We also observed greater occurrence of Brg1-bound enhancers proximal to significantly downregulated genes than expected by chance alone (*P*=0.0002, hypergeometric test). A potential indirect role for *Brg1* in enhancer regulation through transcriptional control of histone modifying enzymes was discounted, as expression of histone acetyltransferases responsible for depositing H3K27ac at enhancers was not affected by loss of *Brg1* (supplementary material Fig. S7). These data support a direct role for *Brg1* in control of enhancer activity.

Enhancer usage is highly cell-type specific and dynamic during cell differentiation, and H3K27ac enrichment distinguishes active enhancers from other enhancer states (Calo and Wysocka, 2013). Given the requirement for *Brg1* for H3K27ac levels at enhancers proximal to downregulated genes and that many of these genes are induced during mesoderm differentiation (Fig. 4A), we hypothesized that *Brg1* might be required to activate quiescent enhancers during mesoderm differentiation. To test this, we used our published enhancer predictions in directed cardiac differentiations of ESCs to distinguish enhancers that are dynamically activated during mesoderm differentiation from those that remain active from undifferentiated cell states (Wamstad et al., 2012). We overlapped our 16,725 predicted enhancers with those identified at an analogous stage of ESC differentiation and divided this cohort into ‘activated’ or ‘static’ enhancers, based on whether these regions were uniquely marked by H3K27ac in mesodermal cultures or marked in both mesodermal cultures and ESCs, respectively. As expected, genes proximal (nearest gene) to activated enhancers are transcriptionally activated during mesoderm induction (data not shown). Whereas static enhancers showed no changes in H3K27ac on average (median log_2_-fold change=0.012), activated enhancers had reduced H3K27ac in *Brg1*-deleted mesoderm (median log_2_ fold change=−1.02) (Fig. 6B,E). Genes proximal to activated enhancers showed greater reductions in gene expression upon loss of *Brg1* than those proximal to static enhancers (Fig. 6F). Moreover, we found that activated enhancers were significantly enriched for Brg1 occupancy compared with static enhancers (Chi-squared test, *P*=3.043e^−11^). Thus, our data reveal that Brg1 activity is most important at regulatory regions that are transitioning in activation status.

Consistent with a role for *Brg1* in activation of mesodermal enhancers, we found multiple Brg1-bound enhancers near the mesodermal genes *Flk1* and *Cyp26a1* that showed marked loss of H3K27ac in *Brg1*-deleted mesoderm (Fig. 6G; supplementary material Fig. S5). This included an experimentally validated regulatory region ∼30 kb upstream of the *Flk1* TSS that directs early mesodermal expression in the mouse embryo (Ishitobi et al., 2011). Furthermore, we detected a clear reduction in H3K27ac within a Brg1-bound region roughly 5 kb upstream of the *Mesp1* TSS, which functions as a regulatory enhancer for *Mesp1* expression (Haraguchi et al., 2001) (Fig. 6G). These enhancers are not marked by H3K27ac in ESCs and, thus, are activated during mesoderm differentiation to coordinate the transcriptional activation of nearby genes. We propose that *Brg1* regulates the transcriptional induction of mesodermal gene expression through binding to distal regulatory regions and facilitating the recruitment of these regions towards the activation of nearby genes.

Finally, Brg1 has been observed to associate with large enhancer collectives that have been dubbed ‘super’ or ‘stretch’ enhancers (Hnisz et al., 2013; Parker et al., 2013; Whyte et al., 2013). Based on correlation with transcriptional activity, these large stretches of H3K27ac have been proposed to be associated with highly cell type-specific gene regulation. We identified 4894 ‘super-enhancers’, of which 594 were bound by Brg1. Although these had significant reductions in H3K27ac occupancy in the absence of Brg1, the loss of H3K27ac was significantly less pronounced than smaller dynamic enhancers (supplementary material Fig. S6). Thus, Brg1 is important for activating initially silent enhancers and is less important at larger enhancers, perhaps due to redundant mechanisms of enhancer activation (Hnisz et al., 2013).

### *Brg1* is required for H3K27me3 at developmental regulators in mesodermal cultures

We next investigated the mechanism by which *Brg1* regulates the repression of developmental regulators in *Brg1*-deleted mesoderm. Intersection of our RNA-seq analysis with published ChIP-seq datasets of H3K27me3 and Polycomb subunit occupancy in undifferentiated ESCs demonstrated that upregulated genes were highly enriched for Polycomb targets (Ku et al., 2008) (supplementary material Fig. S8). Given that *Brg1* positively regulates PRC2 repression of *Hox* loci in undifferentiated ESCs (Ho et al., 2011), we hypothesized that *Brg1* is broadly required for PRC2-mediated silencing in differentiating mesoderm.

**Fig. 7.**
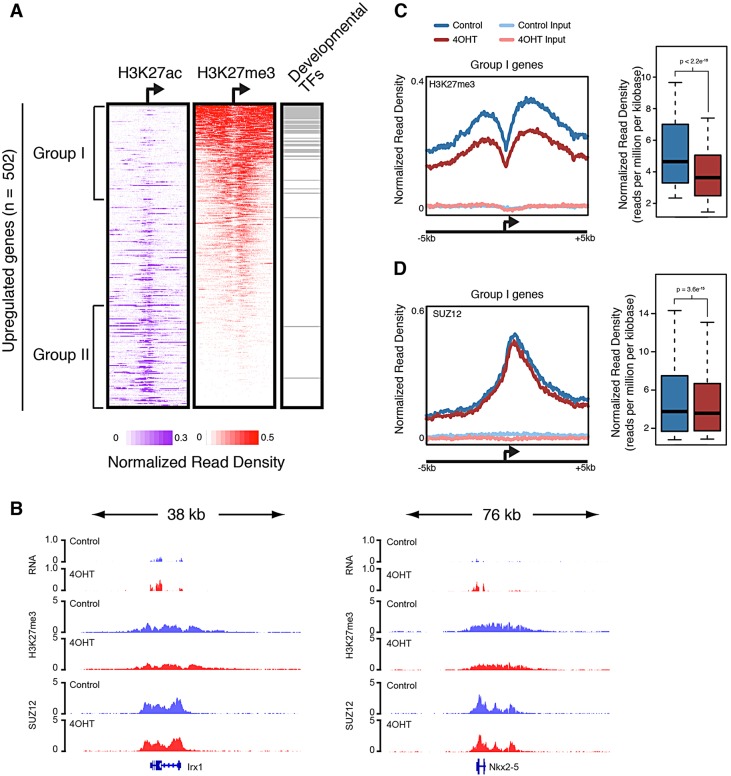
***Brg1* is required for H3K27me3 levels at derepressed developmental regulators.** (A) Density of ChIP-seq reads for H3K27ac and H3K27me3±5 kb of the TSS of upregulated genes. Regions are ranked according to H3K27me3 density. Right bar indicates distribution of developmental transcription factors (TFs). Most developmental TFs are marked by H3K27me3 and not by H3K27ac. (B) Example genomic regions with loss of H3K27me3 at derepressed genes. *y*-axis denotes reads per bin per million. (C,D) (Left) Average ChIP-seq or input signal from control or 4-OHT-treated cultures at promoters of group I genes for (C) H3K27me3 or (D) Suz12. (Right) Box plot of normalized (C) H3K27me3 or (D) Suz12 ChIPseq read density at group I gene promoters (±5 kb of TSS) for day 4 control (blue) or day 4 4-OHT (red). Significance between groups was determined using a two-sided paired Mann–Whitney *U*-test.

To test this, we measured genome-wide occupancy of H3K27me3 in control and 4-OHT-treated mesodermal cultures by ChIP-seq in biological triplicate. We analyzed the enrichment of H3K27me3 at the promoters of genes upregulated by loss of *Brg*1 in normal mesodermal cultures and found that a subset of these genes (termed group I) were substantially enriched for H3K27me3 (Fig. 7A). This subset included nearly all derepressed developmental TFs. In agreement with the exclusivity of the two marks, group I genes were relatively low in H3K27ac, which instead marked a second subset of upregulated genes with few developmental regulators (group II).

We focused on group I genes and compared H3K27me3 genome-wide maps from control and 4-OHT-treated mesodermal cultures, to determine whether loss of *Brg1* led to reduced levels of H3K27me3. Whereas most developmental TFs were still marked by H3K27me3 in Brg1-depleted cultures, we observed clear, reproducible reductions in H3K27me3 at group I genes (Fig. 7B,C; supplementary material Fig. S5C). Clear examples of this are *Irx1* and *Nkx2-5*, two homeodomain TFs that are upregulated upon loss of *Brg1* (Fig. 7B). We did not observe reduced expression of PRC2 subunits or H3K27 demethylases in our RNA-seq analysis, arguing against an indirect effect on H3K27me3 levels through *Brg1* transcriptional regulation of these chromatin regulators (supplementary material Fig. S7). We next considered that reduced levels of H3K27me3 might result from abrogated recruitment of PRC2 or reduced activity of recruited complexes. To distinguish between these two possibilities, we measured genome-wide occupancy of Suz12, an essential subunit of PRC2 complexes (Surface et al., 2010), in control and 4-OHT-treated mesodermal cultures in biological duplicate. Suz12 demonstrated clear enrichment at group I genes in both normal and *Brg1*-deleted cultures. Composite analysis of all group I genes revealed a modest, albeit statistically significant, reduction in Suz12 occupancy (Fig. 7D); however, this reduction was small in comparison to the reduction in H3K27me3. Thus, Brg1 probably modulates PRC2 repression independently of PRC2 recruitment.

## DISCUSSION

**Fig. 8.**
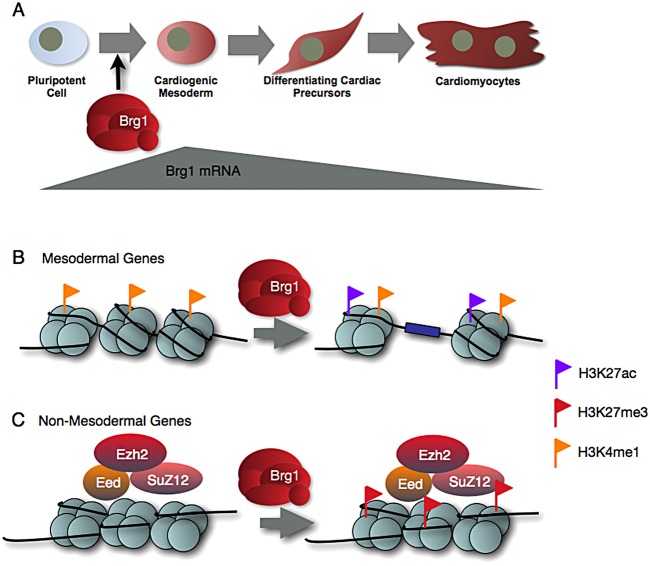
**Summary model.** (A) Dynamic expression of Brg1 during cardiac differentiation peaks at the mesoderm stage. Brg1 function is most crucial during the mesoderm-induction stage. (B,C) Mechanisms of Brg1 function during mesoderm induction. (B) Brg1 is required for silent or poised enhancer to transition to an H3K27ac^+^ active state. (C) Brg1 is required at non-mesodermal genes to promote Polycomb complex-mediated repression.

Our findings support a role for *Brg1* in balancing lineage-specific gene expression (summarized in Fig. 8). In particular, *Brg1* is essential for transcriptional activation of essential mesodermal genes during mesoderm induction. Our genome-wide occupancy data support a primary role for *Brg1* at distal enhancers rather than at promoters. The absence of a strong correlation between Brg1 promoter occupancy and gene regulation might reflect the greater stability of chromatin states at promoter regions seen across cell-types and during differentiation (Ernst et al., 2011; Wamstad et al., 2012).

Brg1-bound loci distal to TSSs largely overlapped putative enhancer regions marked by H3K27ac, consistent with findings in other cell types (Euskirchen et al., 2011; Hu et al., 2011; Rada-Iglesias et al., 2011; Yu et al., 2013). Our data show that H3K27 acetylation depends on Brg1 at a number of loci. Of particular interest, our findings demonstrate that differentiating ESCs are most sensitive to Brg1 function at dynamic enhancer regions, pointing to an essential role for *Brg1* in the transition of developmental enhancers from inactive to active. This might reflect the importance of chromatin remodeling in the conversion of inaccessible chromatin to open chromatin by facilitating TF binding and histone acetyltransferase recruitment. A similar function for BAF complexes has been proposed downstream of Cer1-mediated activation of *Nkx2-5* (Cai et al., 2013). BAF and the related yeast SWI-SNF complexes mediate TF recruitment (Hu et al., 2011; Kwon et al., 1994; Takeuchi and Bruneau, 2009), but the functional interplay between SWI-SNF family complexes and histone acetyltransferases is less clear (Agalioti et al., 2000; Narlikar et al., 2002). Our data suggest that *Brg1* enhances the function of histone acetyltransferases at transitioning enhancers, but the mechanism for this interaction is not clear. Once enhancer regions acquire characteristics of open chromatin, such as H3K27ac, they appear less dependent on *Brg1* in maintaining these characteristics. Thus, our findings predict that, whereas Brg1 might be recruited broadly to enhancer regions in many cell types, *Brg1*-dependent gene expression is likely to reflect those regions undergoing dynamic chromatin remodeling.

*Brg1* is also required for repression of a diverse group of developmental regulators during mesoderm differentiation. BAF complexes have classically been annotated as Trithorax group (TrxG) complexes, which counteract Polycomb-mediated repression, based on studies in *Drosophila* (Tamkun et al., 1992). Indeed, *brm* knockdown leads to increased H3K27me3 in addition to reduced H3K27ac in flies (Tie et al., 2012). In mouse ESCs, loss of *Brg1* is linked to reduced H3K27me3 levels at *Hox* clusters, classic Polycomb targets (Ho et al., 2011). Our study demonstrates that loss of *Brg1* disrupts Polycomb repression of *Hox* clusters, as well as a broad range of other developmental regulators, during ESC differentiation. Thus, cooperativity between PRC2 and BAF complexes is not unique to the pluripotent state and is probably a crucial function for BAF complexes in lineage commitment. The nature of PRC2/BAF cooperativity is unclear. Our ChIP-seq analysis of Brg1 occupancy revealed few clear peaks within H3K27me3-marked domains, whereas Brg1 was found to co-occupy more clearly PRC2-regulated loci in developing organs, including heart (Attanasio et al., 2014). Therefore, in mesoderm Brg1 might associate with Polycomb-repressed genes in rare, transient interactions that are below our threshold for detection. Our ChIP-seq analysis revealed little change in Suz12 occupancy at derepressed genes, suggesting that *Brg1* is dispensable for Suz12 recruitment and might regulate PRC2 activity at bound loci. Nucleosome density affects PRC2 activity *in vitro* (Yuan et al., 2012). Thus, chromatin remodeling by BAF complexes could increase PRC2 efficiency by augmenting nucleosome fluidity. This model requires further exploration.

## MATERIALS AND METHODS

### Cardiomyocyte differentiation

Mouse ESCs were cultured in feeder-free conditions and serum containing media with leukemia inhibitory factor. Directed differentiations were performed as described previously (Wamstad et al., 2012). For Brg1 deletion, *Brg1^fl/fl^*;*Actin-CreER* ESCs (Ho et al., 2009; Ho et al., 2011), cultures were treated with 200 nM 4-hydroxytamoxifen (4-OHT) diluted from a 5 mg/ml stock solution in tetrahydrofuran (THF) or with only THF for control. Additional details are provided in the supplementary material Methods.

### Quantitative PCR

RNA was extracted using TRIzol and reverse transcribed using a High-Capacity cDNA Reverse Transcription kit (Applied Biosystems). Quantitative PCR was performed in technical triplicate using Taqman probes and expression was normalized to *Gapdh*. The following probes were used: *Mesp1* – Mm00801883_g1, *Gapdh* – 4352932E.

### Western blotting, immunofluorescence and FACS analysis

For cell surface staining, cells were trypsinized, quenched with serum and washed in FACS buffer. Cells were stained with biotinylated anti-Flk-1 (Hybridoma Clone D218; 1:10,000) antibody, washed and stained with PE-conjugated anti-Pdgfra (eBioscience, 12-1401-81; 1:400) and APC-Streptavidin (1:200). Cells were analyzed on an LSRII flow cytometer (BD). For intracellular staining, cultures were trypsinized, fixed and stained with anti-cTnT (Thermo Scientific #MS295, Clone 13-11; 1:100) antibody, followed by secondary antibody. All steps were performed in D-PBS with 0.5% saponin and 4% FBS. Western blotting was performed using standard techniques. Briefly, protein lysate was sonicated and cleared by centrifugation. Supernatant was diluted and boiled. Following electrophoresis, protein was transferred to a PVDF membrane. Membranes were incubated with desired antibody in 5% milk TBST overnight at 4°C, then washed in TBST and stained with secondary antibody. After antibody staining, membranes were washed, incubated in SuperSignal chemiluminescence substrate (Thermo Scientific) and visualized. Antibodies used were anti-Brg1 (Santa Cruz, sc-10768; 1:2000), anti-actin (Sigma, A1978; 1:2000) and anti-FLAG (Sigma, M2; 1:2000). For immunofluorescence, cultures were fixed, and, after blocking, were incubated with primary antibody at 4°C overnight. Slides were washed and incubated in secondary antibody. Slides were stained with Hoechst 33342 (10 μg/ml) in D-PBS, and immediately imaged in 50 μl D-PBS. The anti-cTnT (Thermo Scientific #MS295, Clone 13-11; 1:100) antibody was used.

### RNA-seq

Total RNA was isolated from 1.5-2×10^6^ cells using TRIzol reagent in biological duplicates for each experimental condition. 8 μg of total RNA was used as input material for the preparation of the RNA-Seq libraries, according to Illumina RNA Seq library kit with minor modifications. Briefly, mRNA was isolated using Dynabeads mRNA Purification Kit (Invitrogen), followed by fragmentation (Ambion) and ethanol precipitation. First- and second-strand synthesis were performed followed by end repair, A-tailing, adapter ligation and size selection on a Beckman Coulter SPRI TE nucleic acid extractor. 200-400 bp dsDNA was enriched by 13 cycles of PCR with Phusion High-Fidelity DNA Polymerase (NEB). Amplified libraries were sequenced on an Illumina HiSeq 2000.

### RNA-seq analysis

Single-end 40-bp reads were aligned to the mouse genome (mm9) using Bowtie (Langmead et al., 2009). Differential gene expression between conditions was determined using the USeq package (Nix et al., 2008) considering all Refseq genes. Genes with an FDR of ≤1% and twofold expression change were considered significantly differentially expressed unless otherwise noted. USeq was also used to calculate reads per kilobase exon per million reads (RPKM) and fold change values between conditions. Mapped reads were filtered to allow a maximum of 50 identical reads, and genes expressed <0.5 RPKM in all conditions were excluded from subsequent analysis. GO analysis was performed using Go Elite (http://www.genmapp.org/go_elite/), with all genes having an RPKM >0.5 in at least one condition serving as the gene universe. Graphical representation of upregulated and downregulated genes was performed in R.

### ChIP-seq/ChIP-exo

Chromatin immunoprecipitation of histone modifications were performed according to Lee et al. (2006) with minor modifications, in biological duplicate for H3K27ac, Suz12 and FLAG. H3K27me3 ChIP-seq was performed in biological triplicate. Additional details are provided in the supplementary material Methods. Antibodies used were anti-FLAG (Sigma, M2 F1804; 10 µg), anti-H3K27ac (ActiveMotif, #39134; 5 µg), anti-H3K27me3 (Millipore, 17-622; 5 µg) and anti-Suz12 (Bethyl, A302-407A; 5 µg). ChIP-seq analysis pipeline and statistical methods are provided in the supplementary material Methods.

Brg1 ChIP-exo was performed as previously described (Serandour et al., 2013) using anti-Brg1 antibody (Abcam, 110641; 3 µg). Briefly, Brg1 ChIP was performed, and, while still on magnetic beads, the immunoprecipitated DNA was polished, ligated with P7 adapter and nicks were repaired. The resulting DNA was digested with Exo I and RecJ_f_ exonucleases (NEB). Exonuclease-digested DNA was eluted from the beads, cross-links were reversed and the bound protein was digested with proteinase K at 65°C overnight. DNA was purified using Agencourt Ampure XP beads (Beckman Coulter), denatured, and the single-stranded DNA was used to synthesize the second strand using P7 primer, followed by ligation of P5 adapter. The resulting DNA fragment was PCR-amplified, gel-purified and sequenced using an Illumina HiSeq 2500 sequencer at a minimum depth of 25 million mapped reads, with most exceeding 30 million.

### Data deposition

All sequencing data have been deposited in GEO (accession number GSE45448).

## Supplementary Material

Supplementary Material
