# Supplementary material for: Brg1 modulates enhancer activation in mesoderm lineage commitment
Source: Development. 2015 Apr 15;142(8):1418–30. doi: 10.1242/dev.109496 (PMC4392595; doi:10.1242/dev.109496)
Supplement: Supplementary Material [file supp_142_8_1418__index.html]

Supplementary Material 

# Brg1 modulates enhancer activation in mesoderm lineage commitment

## DEV109496 Supplementary Material

**Files in this Data Supplement:**

- Supplementary Material
