# Supplementary material for: Brg1 modulates enhancer activation in mesoderm lineage commitment
Source: Development. 2015 Apr 15;142(8):1418–30. doi: 10.1242/dev.109496 (PMC4392595; doi:10.1242/dev.109496)
Supplement: Supplementary Material [file supp_dev.109496_DEV109496supp.pdf]

Supplementary Material

I Supplemental Methods

Cardiomyocyte differentiation

Mouse ES cells were aggregated into embryoid bodies (EB) and cultured at 75,000 cells/ml for two days in serum free media (3 parts IMDM (Cellgro #15-016-CV): 1 part Ham's F12 (Cellgro #10-080-CV), 0.05% BSA, 2 mM GlutaMax (Gibco), B27 supplement (Gibco #12587010), N2 supplement (Gibco #17502048)) supplemented with 50 ug/ml ascorbic acid and 4.5 x 10^{-4} M monothioglycerol. Embryoid bodies were dissociated and reaggregated for 40 hours in the presence of 5 ng/mL human VEGF (R&D #293-VE) and human Activin A (R&D #338-AC) and human BMP4 (R&D #314-BP) at concentrations empirically determined depending on lot. EBs were dissociated and plated at 470,000 cells/cm^2 in StemPro-34 (Gibco #10639011) supplemented with 2 mM GlutaMax, 50 ug/mL ascorbic acid, 5 ng/mL VEGF, 10 ng/mL human basic FGF (R&D #233-FB) and 25 ng/mL FGF10 (R&D #345-FG) for two days. After two days, StemPro-34 with GlutaMax and ascorbic acid was used and replaced daily.

For Brg1 deletion studies, *Brg1*$^{fl/fl}$;*Actin-CreER* ES cells (Ho et al., 2009, Ho et al., 2011) were cultured and differentiated as described above. Cultures were treated with 200 nM 4-hydroxytamoxifen (4-OHT) diluted from a 5 mg/mL stock solution in tetrahydrofuran (THF) or with only THF for control. Fresh 4-OHT was added with each media change. For cell viability studies, dead cells were identified using Trypan blue.

Flow cytometry

For cell surface staining, cells were briefly trypsinized, quenched with serum, and

washed in FACS Buffer (4% FBS in D-PBS) four times. After washing, cells were stained with a biotinylated anti-FLK-1 (Hybridoma Clone D218) antibody for 30 minutes at 4°C. Cells were then washed three times in FACS Buffer and stained with a PE-conjugated anti-PDGFRα (eBioscience 12-1401-81, 1:400) and APC-Streptavidin (1:200) for 30 minutes at 4°C. Cells were three times in FACS Buffer and analyzed on an LSRII flow cytometer (BD). Data was analyzed using FlowJo software (Treestar).

For intracellular staining, cultures were trypsinized, quenched with serum, and fixed in D-PBS with 3.7% formaldehyde for 30 minutes at room temperature. Fixed samples were washed twice and stained with anti-cTnT (Thermo Scientific #MS295, Clone 13-11). antibody. After staining, samples were washed twice, incubated with secondary antibody, and washed two additional times. All steps performed in D-PBS with 0.5% saponin and 4% FBS. Samples were stained with Hoechst 33342 (10 ug/mL) in D-PBS with 4% FBS. Samples were analyzed as above.

Quantitative PCR

RNA was extracted using TRIzol*®* and reversed transcribed using High-Capacity cDNA Reverse Transcription kit (Applied). Quantitative PCR was performed in technical triplicate using Taqman probes and expression was normalized to *Gapdh*. The following probes were used: *Mesp1* – Mm00801883_g1, *Gapdh* – 4352932E

Western blotting

Western blotting was performed using standard techniques. Briefly, protein lysate was sonicated (4 pulses for 30 seconds) and cleared by centrifugation at 13,000 RPM. Supernatant was diluted 1:1 with 2x Laemmli Buffer and 100 mM DTT and boiled for 10 minutes at 95°C. Following electrophoresis, protein was transferred to a PVDF

membrane. Membranes were blocked for 1 hour at room temperature with 5% milk Tris-buffered saline Tween (TBST). Following blocking, membranes were incubated with desired antibody in 5% milk TBST overnight at 4°C. Membranes were washed 4 times for 15 minutes at room temperature in TBST and then stained with secondary antibody in 5% milk TBST for 1 hour at room temperature. After antibody staining, membranes were washed as after primary incubation, incubated in SuperSignal chemiluminescence substrate (Thermo Scientific), and visualized. Antibodies used were anti-BRG1 (Santa Cruz sc-10768), anti-actin (Sigma A1978), and anti-FLAG (Sigma M2).

Immunofluorescence

Cultures were fixed for 30 minutes at room temperature in 3.7% formaldehyde D-PBS and washed once with D-PBS. Wells were blocked in 2% bovine serum albumin 0.1% Triton-X-100 D-PBS for 30 minutes at RT. After blocking, cultures were incubated with primary antibody at 4°C overnight. Slides were washed three times with 0.1% Triton X-100 D-PBS and incubated in secondary antibody at room temperature for 1 hour. After staining, slides were washed three times with 0.1% Triton X-100 D-PBS, stained with Hoechst 33342 (10 ug/mL) in D-PBS, and immediately imaged in 50 uL D-PBS. Antibodies used were anti-cardiac isoform of Troponin T (cTnT) 1:100 (Thermo Scientific #MS295, Clone 13-11).

Transfections

HeLa cells were transfected with 1 ug of plasmid using X-tremeGene (Roche) and cells were harvested 42 hrs after transfection.

ChIP-Seq / ChIP-exo

Frozen pellets of cross-linked cells ($10x10^6$) were thawed in cold lysis buffer 1 (50 mM HEPES-KOH, pH 7.5, 140 mM NaCl, 1 mM EDTA, 10% glycerol, 0.5% NP-40, 0.25% Triton X-100, 1× protease inhibitors) and gently rocked at 4°C for 10 minutes in 15 mL conical tubes. Cells were pelleted at 1350 x g at 4°C and resuspended in cold lysis buffer 2 (10 mM Tris-HCl, pH 8.0, 200 mM NaCl, 1 mM EDTA, 0.5 mM EGTA, 1× protease inhibitors) and gently rocked at 4°C for 10 minutes in 15 mL conical tubes. Cells were pelleted at 1350 x g at 4°C in a table top centrifuge and resuspended in 0.5 mL cold ChIP lysis buffer (50 mM HEPES-NaOH, pH 7.5, 140 mM NaCl, 1 mM EDTA, 1% Triton X-100, 0.1% SDS, 0.1% sodium deoxycholate) and sonicated to 200-1000 bp fragments using a VirSonic sonicator. Sonicated lysates were cleared by pelleting insoluble material at 13,000 RPM at 4°C followed by incubation with 5 ug antibody overnight. Next, Protein A magnetic beads (45 uL) were added to the lysate and incubated at 4°C for 7 hrs. Prior to addition, magnetic beads were washed 3 times with block (0.5% BSA/PBS). Immunoprecipitated material was washed 2 times each with ChIP lysis buffer, high salt lysis buffer (50 mM HEPES-NaOH pH 7.5, 500 mM NaCl, 1 mM EDTA, 1% Triton X-100, 0.1% SDS, 0.1% sodium deoxycholate), and LiCl wash buffer (10 mM Tris-HCl pH 8.0, 250 mM LiCl, 1 mM EDTA, 0.5% NP-40, 0.5% sodium deoxycholate) and one time with TE plus NaCl, followed by elution and reverse crosslinking in 210 uL of 1% SDS in TE overnight at 65°C. 200 uL of uncrosslinked material was treated with RNase A for 2 hours, proteinase K for 2 hours, and extracted 2 times with phenol/chloroform/isoamyl alcohol. This was followed by ethanol precipitation with a glycogen coprecipitant, 80% ethanol wash and final resuspension in TE. Nucleic acid yield was determined via PicoGreen (Invitrogen). Adapter ligation and size selection (200-400 bp) were performed using a Beckman Coulter SPRI TE nucleic acid extractor,

or, in some cases, performed by hand. Fragments were PCR amplified for 13 cycles followed by sequencing on an Illumina HiSeq 2000 system. For hand prepared libraries, libraries were made using the Ovation Library Prep kit according the manufacturer's instructions (NuGen). Briefly, DNA samples were end repaired, ligated to adaptors, and purified using AMPure beads. Purified samples were amplified by PCR for 17 cycles, purified, and sequenced on an Illumina HiSeq 2000 system.

For BRG1-FLAG ChIP-seq, the protocol described above was followed except 8-10x10^6 cells were used as starting material and 10 ug antibody was incubated with Protein G magnetic beads for roughly 6 hours prior to washing and addition of the bead/antibody complex to chromatin lysate for immunoprecipitation overnight.

Brg1 ChIP-exo was performed as previously described (Serandour et al., 2013) using anti-BRG1 antibody (1mg, Abcam 110641). ChIPExo was performed on Brg1 ChIP material while still on protein G magnetic dynabeads. After incubation with the antibody, the beads were washed six times with RIPA buffer (50mM HEPES, pH7.6, 1mM EDTA, pH8.0, 0.7% Sodium deoxycholate, 1% NP-40 and 500mM Lithium chloride) and two times with Tris (10mM Tris.Cl, pH 8.0). The bead bound DNA were end polished at 30°C for 30mins in using T4 DNA polymerase, Klenow fragment of DNA polymerase and T4 polynucleotide kinase. From this point each subsequent enzymatic steps were followed by two washes with each of RIPA and 10mM Tris. P7 adapter was ligated to the DNA ends using T4 DNA ligase at 25°C for 60 mins followed by nick repair using Phi29 polymerase at 30°C for 20 mins. Samples were periodically vortexed at 900rpm in a thermomixure during enzymatic reaction. DNA was digested with λ and RecJf exonucleases at 37°C for 30 mins each. Samples were then eluted off the beads with 100μl of Elution buffer (50mM Tris.Cl, pH 8.0, 10mM EDTA, 1% SDS) by incubating at

65°C for 30mins with periodic shaking. RNase A was added to the samples for 30min at 37°C. Proteinase K was added to degrade proteins, crosslink was reversed by overnight incubation at 65°C and using Ampure beads ChIP DNA was purified. The purified DNA was denatured at 95°C for 5 mins before synthesis of the 2nd strand by P7 primer extension in presence of Phi29 polymerase. Then, P5 adapters were ligated to the DNA ends and the DNA fragments were PCR amplified for 18 cycles using universal primers containing the index sequences. PCR products were purified using Ampure beads, size selected using 2% agarose gels in an E-Gel Electrophoresis system (Invitrogen), gel purified using minielute gel extraction columns (Qiagen) and eluted in 20μl of TE. Samples were quantified and analysed on Qubit and Bioanalyser before sequencing on a Illumina HiSeq 2500 sequencing machine.

ChIP-seq/ChIP-exo analysis pipeline

Single end 40 bp reads were aligned to the mouse genome (mm9) using Bowtie (Langmead et al., 2009). Unique sequences were extended +200 bp and allocated in 25-bp bins. Input DNA was used as a background model. A Poissonian model was used to determine statistically enriched bins with a P-value threshold set at $1x10^{-12}$ for H3K27me3, H3K27ac, and Suz12 and $1x10^{-6}$ for Flag-Brg1 as described previously (Marson et al., 2008). Genomic browser tracks were generated using the Integrated Genome Viewer (Robinson et al., 2011). Browser tracks and other downstream analysis was performed on pooled data from multiple replicates. Similar trends were observed if replicates were analyzed individually.

Computational analysis

Chromatin regulator expression

To visualize expression patterns for annotated chromatin regulators, chromatin regulators annotated as involved in chromatin remodeling (GO0006338) or covalent chromatin modification (GO00016569) were considered. Genes *Smarca2*, *Pbrm1*, *Arid1b*, *Smarcd3*, *Smarcd2*, *Phf10*, *Dpf1*, *Dpf2*, *Dpf3*, *Smarce1*, *Arid2*, and *Arid1a* were manually added to this list based on the literature. The median expression values of these genes for four stages of cardiac differentiation (Wamstad et al., 2012) were median centered and interquartile range scaled and clustered using the bioconductor package Hopach (http://www.bioconductor.org/packages/2.1/bioc/html/hopach.html) and a cosine angle distance metric.

Tissue and cell type expression of upregulated developmental TFs

Normalized expression data from the Gene Atlas GNF1M dataset (Su et al., 2004) for upregulated developmental TFs were averaged (mean) between replicates and visualized as a heatmap. Developmental TFs were identified as genes that are annotated as within both GO terms GO0003700 (sequence-specific DNA binding transcription factor activity) and GO0048856 (anatomical structure development).

Classification of BRG1 peaks, enhancers, and H3K27me3 domains

To identify BRG1 bound regions, we overlapped statistically enriched peaks over two replicates of FLAG ChIP-seq and required peaks in both replicates to be within 1 kb. Calling peak overlap within 500 bp reduces the number of called peaks by less than 10%, and not much more by calls within 250 bp, indicating that concordant peaks overlap well. Peaks conserved across replicates were then merged into a single region that included both peaks and any genomic space in between. In order to classify genomic localization of BRG1 bound regions, Ensembl lists of genes and exons were

intersected with BRG1 enriched regions. Promoter regions were classified as ±2.5 kb of the TSS. If a BRG1 bound region overlapped with more than one classification, it was labeled according to the following order: Promoter > Exon > Intron > Intergenic. Thus all peaks that did not overlap with promoters, exons, or introns were labeled intergenic. Putative enhancer regions were identified by intersecting regions of H3K27ac enrichment for both biological replicates. The size of the enhancer represents the combined length of the enriched regions for each replicate. Blocks of genomic space ± 2.5 kb from the Ensembl TSSs were subtracted to yield a list of high-confidence putative distal enhancers. To determine the proportion of Brg1 peaks that fall within putative enhancers, we intersected these regions, requiring at least one base pair of overlap.

To identify enhancers associated with blocks labeled "super" enhancers, we utilized the ROSE (https:// bitbucket.org/young_computation/rose) algorithm that has been previously described (Hnisz et al., 2013). Briefly, enhancers within 12.5 kb were merged and ranked according to input-subtracted signal of H3K27ac, which is used to determine a H3K27ac signal inflection point and identify super enhancers. Enhancers within ± 2.5 kb of the TSS were excluded from this analysis. These domains of "super" enhancers were then intersected with all identified enhancers to identify enhancers associated with super enhancer domains.

Motif enrichment within BRG1+ enhancers was done by scanning the subset of BRG1-bound regions that completely overlapped with a putative enhancer using TRANSFAC's 'match' algorithm for whether or not each vertebrate motif in the database was identified within 75 bp of the center of that enriched region, and the hypergeometric distribution was used to calculate the probability that a given number of each motif would be seen among BRG1-associated peaks, given its abundance among all H3K27-acetylated

enhancers, and the false discovery rates for these probabilities were controlled using the q-value (Storey and Tibshirani, 2003). Each enriched motif was annotated with the expression at each stage of in vitro cardiomyocyte differentiation using data from (Wamstad et al., 2012).

Replicate Correlation

Unique sequence were extended by 200 bp and allocated into 20 bp bins. Bin read density was calculated and compared across all genomic bins using a Spearman's rank correlation.

Visualization of ChIP-seq data

To determine the average profile of H3K27me3, H3K27ac, or SUZ12 ChIP-seq and Input signal around transcriptional start sites or enhancer regions, unique aligned reads within 5 kb of the TSS or the midpoint of enhancer regions were grouped into 50 bp bins and then normalized based on number of reads per library (reads per million). In cases where the ChIP-seq read density of many promoters or regions of interest were visualized together, normalized binned read counts for each gene were visualized as heatmaps using R. For boxplots, a normalized read count was computed ±5 kb from the TSS. For boxplots for enhancer regions, a $\log_2$ fold change in normalized read count was computed between conditions for each predicted enhancer region or subset of enhancers investigated.

Polycomb target analysis

TSSs were extended 1 kb in each direction and compared to genome-wide data sets for H3K27me3 and Polycomb subunits in ES cells (Ku et al., 2008) to identify overlap.

Transcriptional start sites for all Refseq genes and genes corresponding to these significantly upregulated or downregulated based on the RNA-seq analysis were considered. A hypergeometric test was used to determine statistical significance and calculate p-values for this comparison.

Statistical Analysis

To look for statistical enrichment of BRG1 and enhancer overlap, we generated 10,000 lists of randomly generated enhancer regions of identical size to our experimentally-derived list. We then intersected each random list with BRG1 bound regions to generate a normal distribution of expected overlap. P-value represents the number of permutations over our random enhancer lists with equal or more overlap than our experimental list.

II. Supplementary Datasets

Supplemental Table 1.

Median centered and interquartile range scaled RPKM expression values for chromatin regulators during directed cardiomyocyte differentiation of embryonic stem cells (Wamstad et al. 2012). Chromatin regulators are genes found in GO categories GO0006338 and GO00016569 and other manually curated genes.

Supplemental Table 2.

Differential gene expression between Day4 4OHT and Day4 Control treated Brg1f/f; Actin-CreER ESCs. Gene list is filtered to remove any genes not expressed above 0.5 RPKM in either condition. Includes genes significantly downregulated or upregulated at a false discovery rate of 1% and three fold change cutoffs (1.2x, 1.5x, and 2.0x).

Supplemental Table 3.

Enriched Gene Ontology terms for significantly downregulated and upregulated genes. Tables are standard output for GOElite.

Supplemental Table 4.

Putative enhancer list: Predicted enhancer regions in mesodermal cultures based on H3K27ac enrichment. Table includes genomic coordinates for each putative enhancer region and the gene with the closest transcriptional start site.

Brg1 enriched regions: Table with the genomic coordinates of BRG1 enriched regions in mesodermal cultures. Brg1 Enriched Regions are the combined length of Brg1 peaks within 1 kb of each other in both replicates from BRG1-FLAG mESCs, including genomic sequence between the peaks.

III. Supplemental Figure Legends

Fig S1. Loss of *Brg1* during mesoderm differentiation reduces viable cells in cardiogenic conditions. (A) Phase contrast images of *Brg1* f/f; Actin-CreER ESCs at Day 11 of directed differentiation demonstrates reduced cell density in cultures treated with 4-OHT at Day 2. Scale bar is 50 μm. (B) Number of viable cells in cultures treated with control or 4-OHT at Day 2 after replating at 150,000 cells per well. Solid and dashed lines indicate two separate differentiations. (C) Treatment of the control ESC line E14 Tg(Nkx2-5-EmGFP) that lacks the Actin-CreER transgene differentiates with comparable efficiency across a range of BMP4 when treated with vehicle control or 200 nM 4-OHT beginning at Day 2 of differentiation. cTnT % was determined by intracellular FACS at Day 10. Three independent differentiations are shown.

Fig S2. Developmental transcription factors upregulated by loss of *Brg1* in mesoderm function in diverse development lineages. Expression levels of 72 developmental transcription factors that are upregulated by loss of *Brg1* in various mouse tissues and cell types. Samples are grouped based on similarities in embryonic origin or function.

Fig S3. Genes upregulated by loss of *Brg1* in mesoderm are lowly expressed in normal mesoderm cultures. Upregulated genes were binned based on their expression decile in normal mesoderm cultures (Day 4 control RNA-seq).

Fig S4. Characterization of *Brg1*-Flag embryonic stem cells. (A) Western blot of protein lysate from *Brg1*-Flag ESC demonstrates robust expression of BRG1-Flag protein. HeLa cells transfected with Flag- and myc- tagged expression constructs serve as positive and negative controls, respectively. (B) Immunoprecipitation of BRG1-Flag from *Brg1*-Flag ESC protein lysate using an anti-Flag antibody and visualized by silver stain. BRG1-Flag co-immunoprecipitates with multiple proteins with molecular weights consistent with known BAF complex components. An immunoprecipitation in the NkxGFP ESC line, which does not express BRG1-Flag, does not pull down these proteins.

Fig S5. Examples of histone modification changes found at misregulated genes in Brg1-deficient mesoderm cultures. (A,B) Tracks for RNA-seq and H3K27ac, H3K27me3, SUZ12, Flag, and Brg1 ChIP-seq/ChIP-exo are shown for genomic regions flanking dysregulated genes. (A) BRG1 binding at putative enhancers proximal to downregulated genes. BRG1 bound enhancers show reduced H3K27ac. (B) Example derepressed genes that demonstrate reduced levels of H3K27me3. Control samples are colored blue and 4-OHT samples are colored red. Tan boxes indicate regions of BRG1 enrichment.

Fig S6. *Brg1* does not affect global levels of H3K27ac at promoters. (**A**) Histogram of $\log_2$ fold change in H3K27ac at Refgene promoters. (**B**) Boxplots of $\log_2$ fold change of subsets of Refgene promoter. N indicates the number of promoters include in each set. (**C**) Boxplots of $\log_2$ fold change of subsets of predicted enhancers. Enhancers are separated into Brg1 bound and unbound cohorts based on the presence or absence of a Brg1 peak respectively. These groups were subdivided into static enhancers (enhancers found in both embryonic stem cells and mesodermal cultures), activated enhancers (enhancers found only in mesodermal cultures), and "super enhancers" (predicted using methods described in (Hnisz et al., 2013)). BRG1 bound, activated enhancers show the greatest average loss in H3K27ac. "Super" enhancers bound by BRG1 had a more modest reduction in H3K27ac than BRG1-bound activated enhancers. N indicates the number of enhancers included in each set. Enhancers and promoters that gave undefined fold change values were excluded. All comparisons significant at $P<0.00001$ (by two-sided KS test).

Fig S7. RPKM values of Polycomb subunits or other H3K27-modifying enzymes in control or 4-OHT treated mesoderm cultures. Loss of *Brg1* does not affect RNA expression of these genes.

Fig S8. Upregulated genes are repressed by Polycomb repressive complexes in embryonic stem cells. Percent of all genes or those upregulated by loss of Brg1 in mesoderm that are marked by H3K27me3 or bound by SUZ12 in ES cells (Ku et al. 2008). Upregulated genes demonstrate significant enrichment for both groups. * $p = 9.03\times10^{-80}$; ** $p=5.15\times10^{-82}$

Supplementary Methods References

Hnisz, D., Abraham, B. J., Lee, T. I., Lau, A., Saint-Andre, V., Sigova, A. A., Hoke, H. A. and Young, R. A. (2013). Super-Enhancers in the Control of Cell Identity and Disease. *Cell*.

Ku, M., Koche, R. P., Rheinbay, E., Mendenhall, E. M., Endoh, M., Mikkelsen, T. S., Presser, A., Nusbaum, C., Xie, X., Chi, A. S., et al. (2008). Genomewide analysis of PRC1 and PRC2 occupancy identifies two classes of bivalent domains. *PLoS Genet* **4**, e1000242.

Langmead, B., Trapnell, C., Pop, M. and Salzberg, S. L. (2009). Ultrafast and memory-efficient alignment of short DNA sequences to the human genome. *Genome Biol* **10**, R25.

Marson, A., Levine, S. S., Cole, M. F., Frampton, G. M., Brambrink, T., Johnstone, S., Guenther, M. G., Johnston, W. K., Wernig, M., Newman, J., et al. (2008). Connecting microRNA genes to the core transcriptional regulatory circuitry of embryonic stem cells. *Cell* **134**, 521-533.

Robinson, J. T., Thorvaldsdottir, H., Winckler, W., Guttman, M., Lander, E. S., Getz, G. and Mesirov, J. P. (2011). Integrative genomics viewer. *Nat Biotechnol* **29**, 24-26.

Serandour, A. A., Brown, G. D., Cohen, J. D. and Carroll, J. S. (2013). Development of an Illumina-based ChIP-exonuclease method provides insight into FoxA1-DNA binding properties. *Genome Biol* **14**, R147.

Storey, J. D. and Tibshirani, R. (2003). Statistical significance for genomewide studies. *Proc Natl Acad Sci U S A* **100**, 9440-9445.

Su, A. I., Wiltshire, T., Batalov, S., Lapp, H., Ching, K. A., Block, D., Zhang, J., Soden, R., Hayakawa, M., Kreiman, G., et al. (2004). A gene atlas of the mouse and human protein-encoding transcriptomes. *Proc Natl Acad Sci U S A* **101**, 6062-6067.

Wamstad, J. A., Alexander, J. M., Truty, R. M., Shrikumar, A., Li, F., Eilertson, K. E., Ding, H., Wylie, J. N., Pico, A. R., Capra, J. A., et al. (2012). Dynamic and coordinated epigenetic regulation of developmental transitions in the cardiac lineage. *Cell* **151**, 206-220.

Supplemental Figures

Fig S1

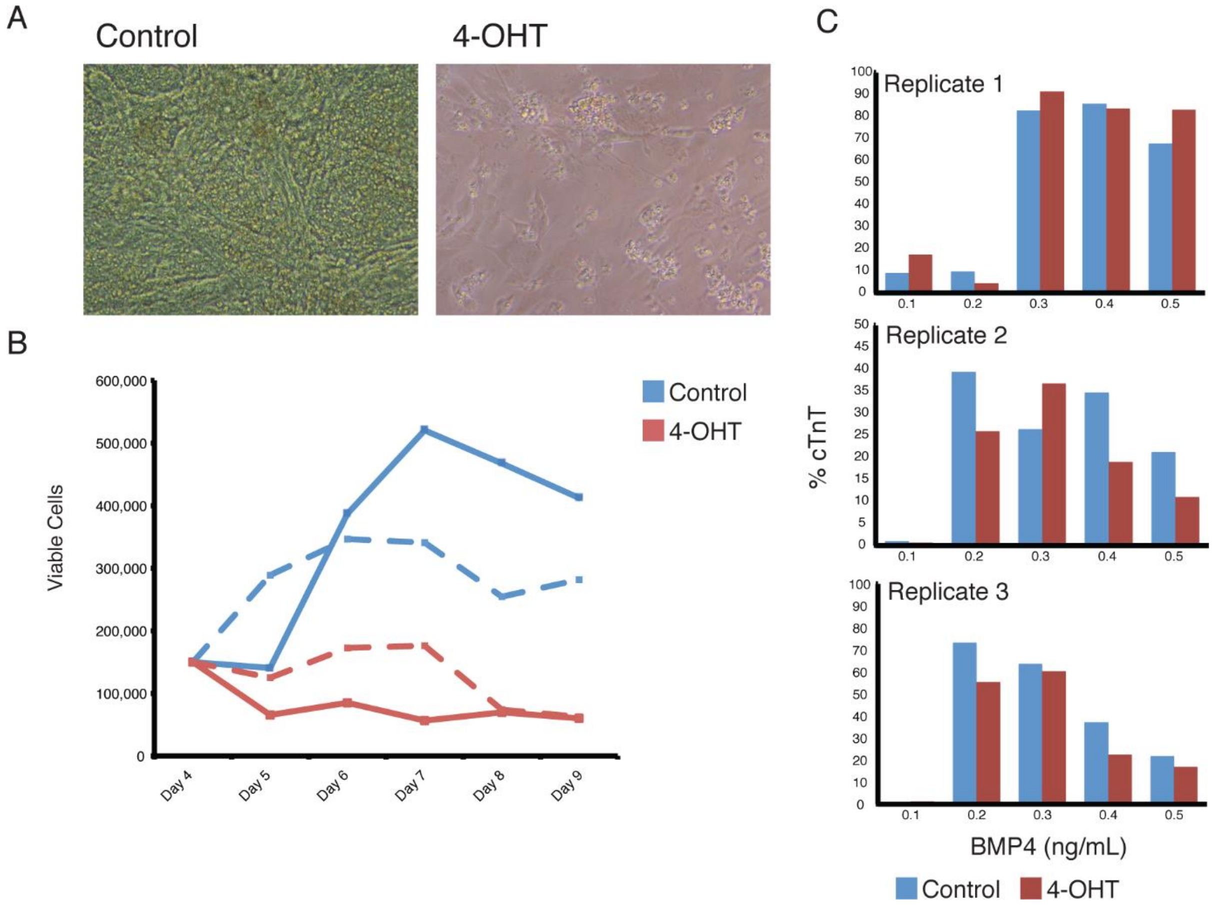

Fig S2

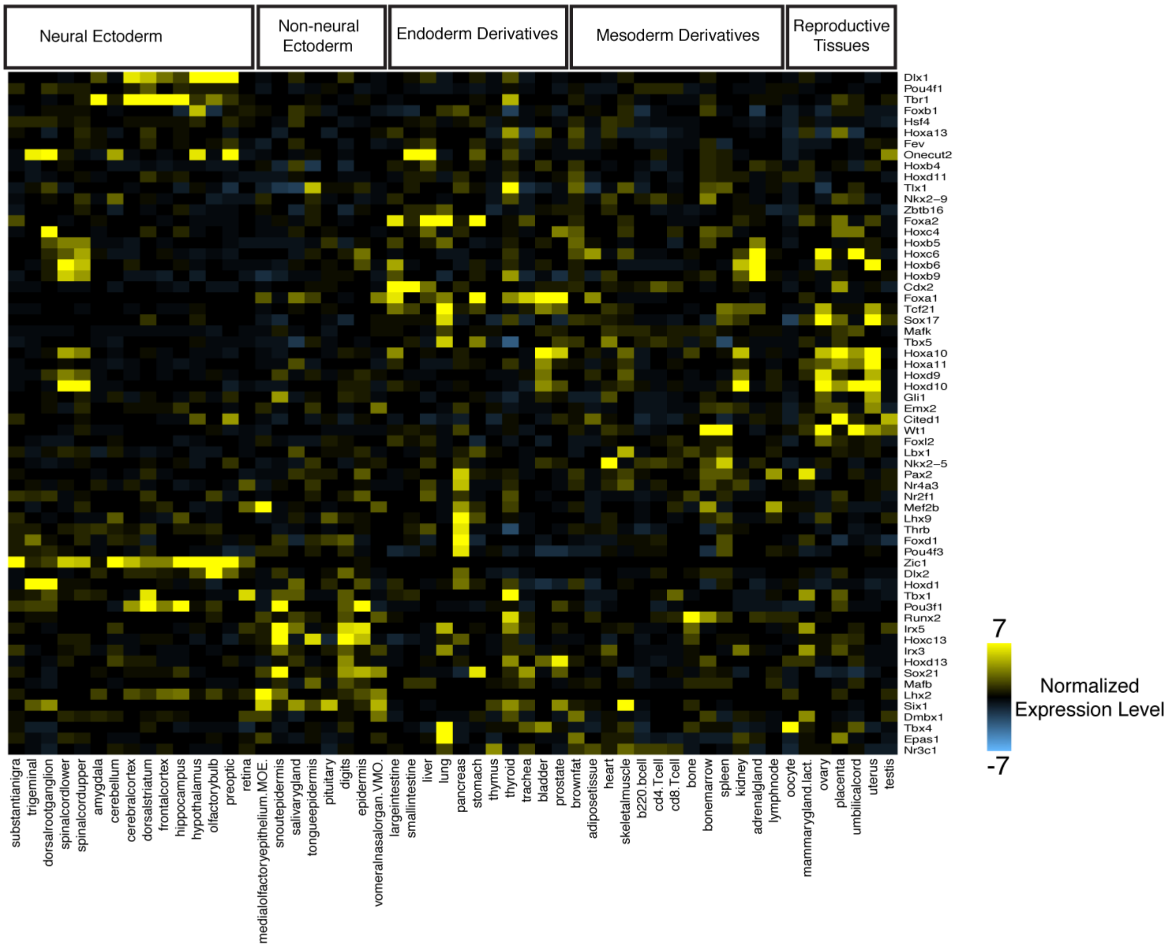
Neural Ectoderm
Non-neural Ectoderm
Endoderm Derivatives
Mesoderm Derivatives
Reproductive Tissues
Dlx1
Pou4f1
Tbr1
Foxb1
Hsf4
Hoxa13
Fev
Onecut2
Hoxb4
Hoxd11
Tlx1
Nkx2–9
Zbtb16
Foxa2
Hoxc4
Hoxb5
Hoxc6
Hoxb6
Hoxb9
Cdx2
Foxa1
Tcf21
Sox17
Mafk
Tbx5
Hoxa10
Hoxa11
Hoxd9
Hoxd10
Gli1
Emx2
Cited1
Wt1
Foxl2
Lbx1
Nkx2–5
Pax2
Nr4a3
Nr2f1
Mef2b
Lhx9
Thrb
Foxd1
Pou4f3
Zic1
Dlx2
Hoxd1
Tbx1
Pou3f1
Runx2
Irx5
Hoxc13
Irx3
Hoxd13
Sox21
Mafb
Lhx2
Six1
Dmbx1
Tbx4
Epas1
Nr3c1
substantianigra
trigeminal
dorsalrootganglion
spinalcordlower
spinalcordupper
amygdala
cerebellum
cerebralcortex
dorsalstriatum
frontalcortex
hippocampus
hypothalamus
olfactorybulb
preoptic
retina
medialolfactoryepithelium.MOE.
snoutepidermis
salivarygland
tongueepidermis
pituitary
digits
epidermis
vomeralnasalorgan.VMO.
largeintestine
smallintestine
liver
lung
pancreas
stomach
thymus
thyroid
trachea
bladder
prostate
brownfat
adiposetissue
heart
skeletalmuscle
b220.bcell
cd4.Tcell
cd8.Tcell
bone
bonemarrow
spleen
kidney
adrenalgland
lymphnode
oocyte
mammarygland.lact.
ovary
placenta
umbilicalcord
uterus
testis
7
Normalized Expression Level
-7

Fig S3

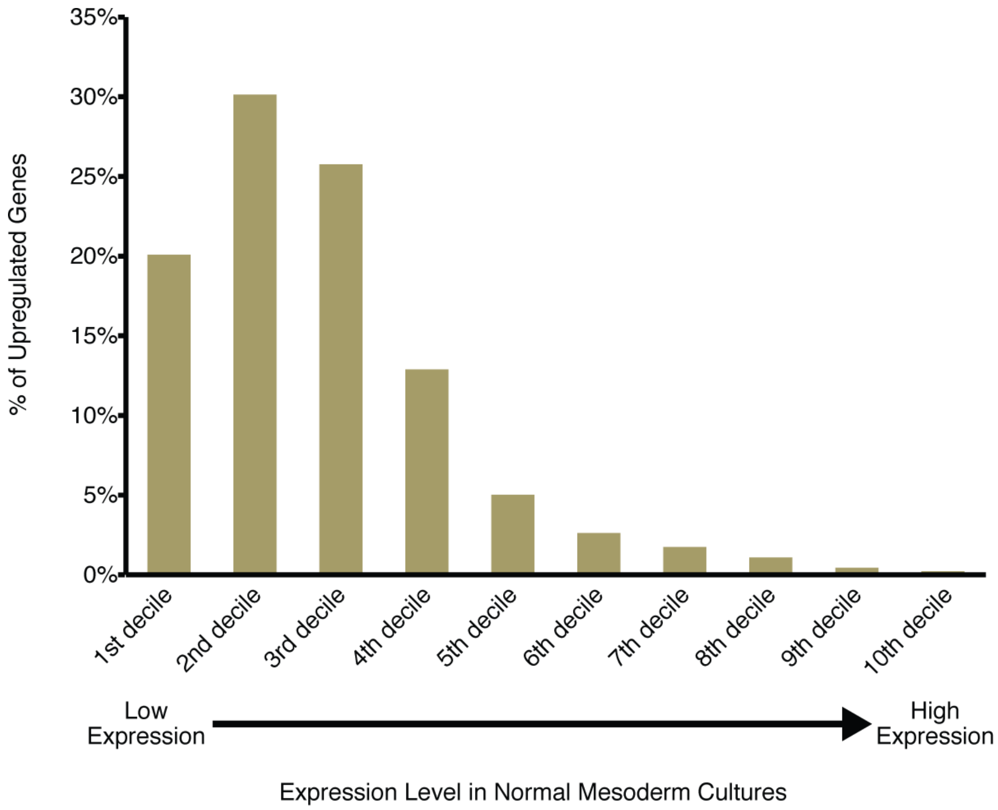
35%
30%
25%
20%
15%
10%
5%
0%
% of Upregulated Genes
1st decile
2nd decile
3rd decile
4th decile
5th decile
6th decile
7th decile
8th decile
9th decile
10th decile
Low Expression
High Expression
Expression Level in Normal Mesoderm Cultures

Fig S4

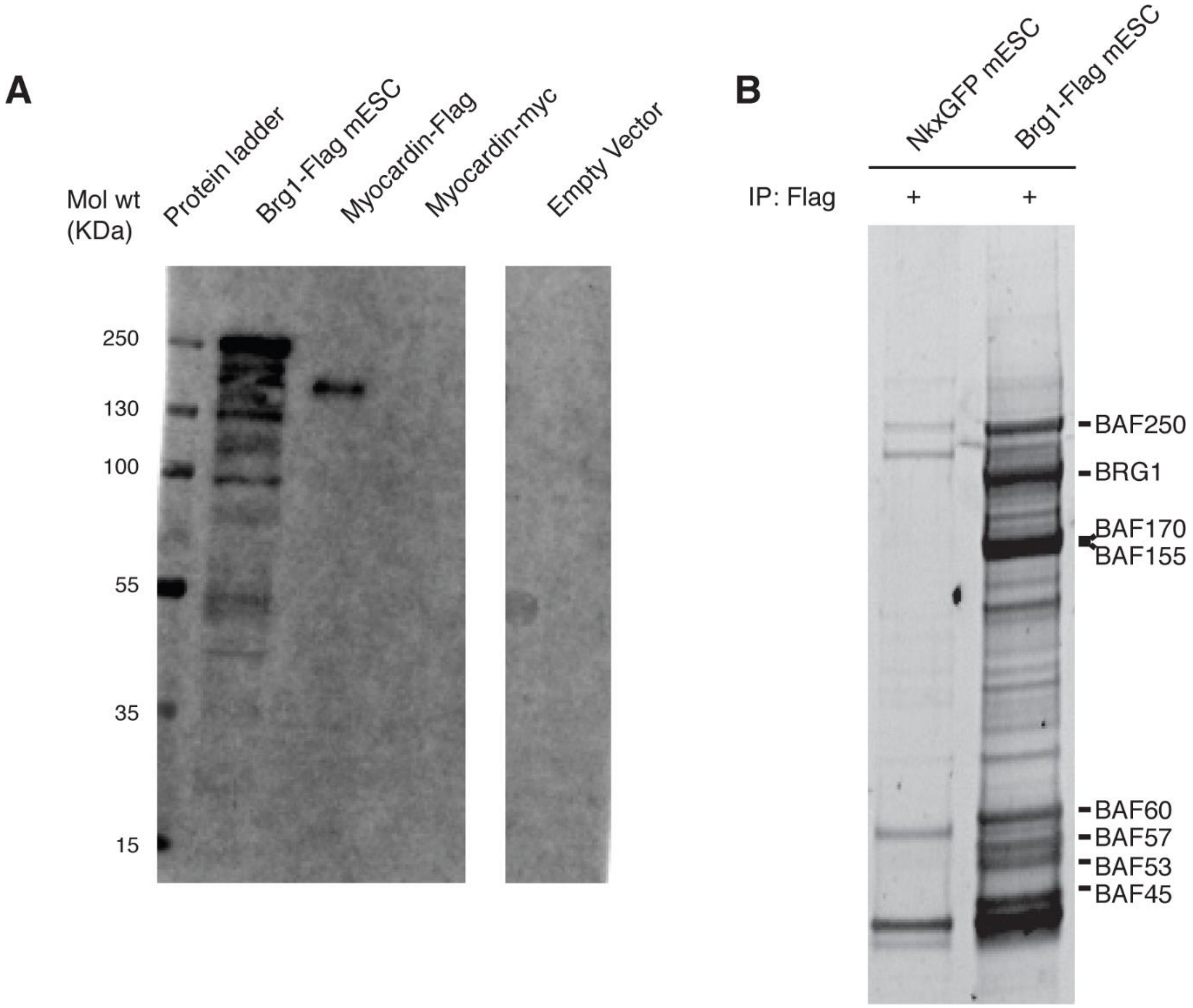
A
Mol wt
(KDa)
Protein ladder
Brg1-Flag mESC
Myocardin-Flag
Myocardin-myc
Empty Vector
250
130
100
55
35
15
B
NkxGFP mESC
Brg1-Flag mESC
IP: Flag
+
+
BAF250
BRG1
BAF170
BAF155
BAF60
BAF57
BAF53
BAF45

Fig S5

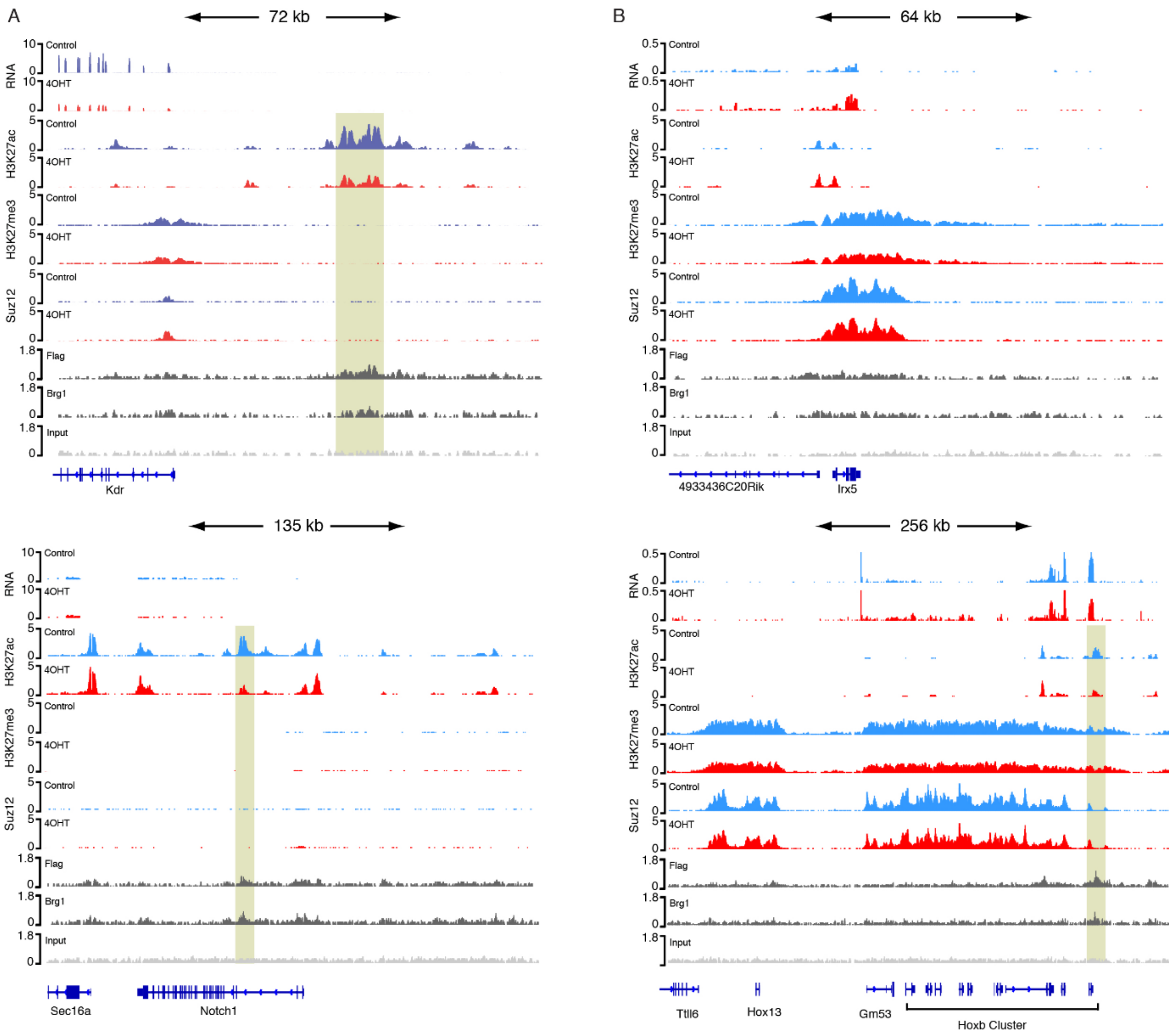
A
72 kb
RNA
H3K27ac
H3K27me3
Suz12
Control
4OHT
Flag
Brg1
Input
Kdr
B
64 kb
4933436C20Rik
Irx5
135 kb
Sec16a
Notch1
256 kb
Ttll6
Hox13
Gm53
Hoxb Cluster

Fig S6

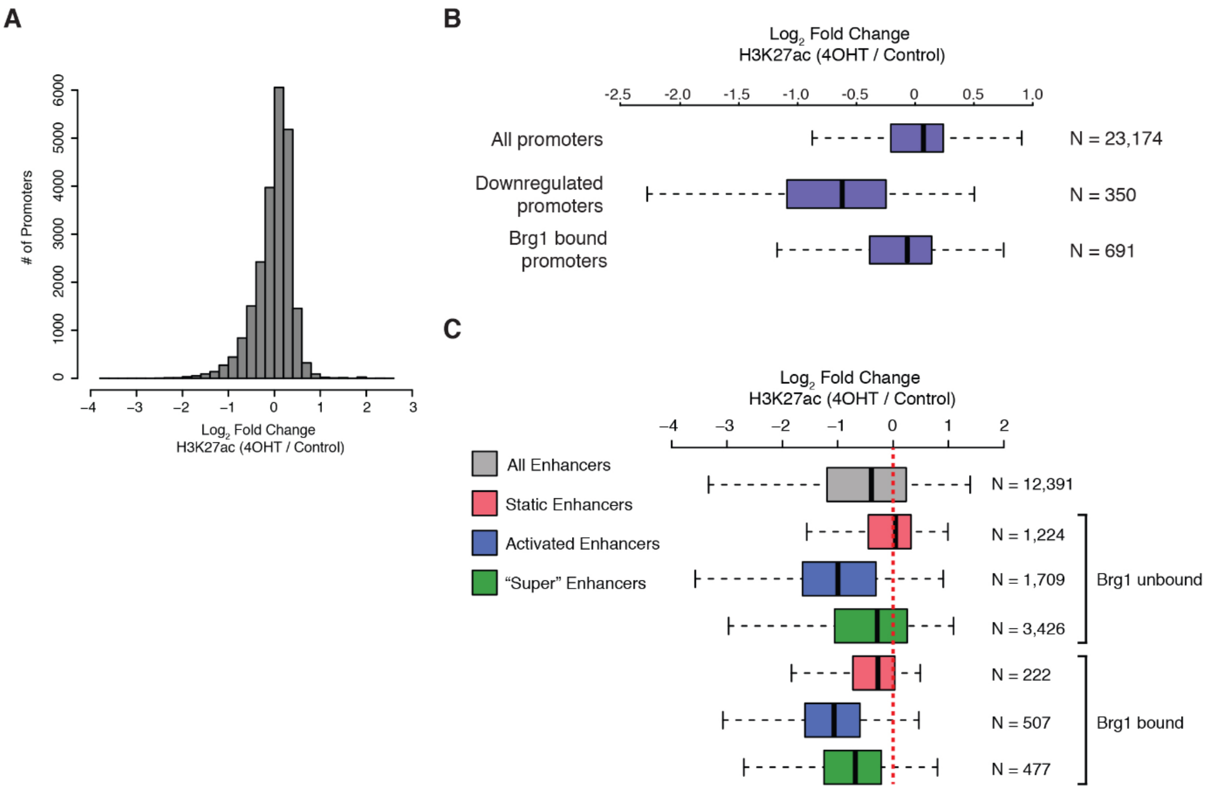
A
# of Promoters
0
1000
2000
3000
4000
5000
6000
–4
–3
–2
–1
0
1
2
3
Log2 Fold Change
H3K27ac (4OHT / Control)
B
Log2 Fold Change
H3K27ac (4OHT / Control)
-2.5
-2.0
-1.5
-1.0
-0.5
0
0.5
1.0
All promoters
N = 23,174
Downregulated promoters
N = 350
Brg1 bound promoters
N = 691
C
Log2 Fold Change
H3K27ac (4OHT / Control)
–4
–3
–2
–1
0
1
2
All Enhancers
Static Enhancers
Activated Enhancers
"Super" Enhancers
N = 12,391
N = 1,224
N = 1,709
N = 3,426
Brg1 unbound
N = 222
N = 507
N = 477
Brg1 bound

Fig S7

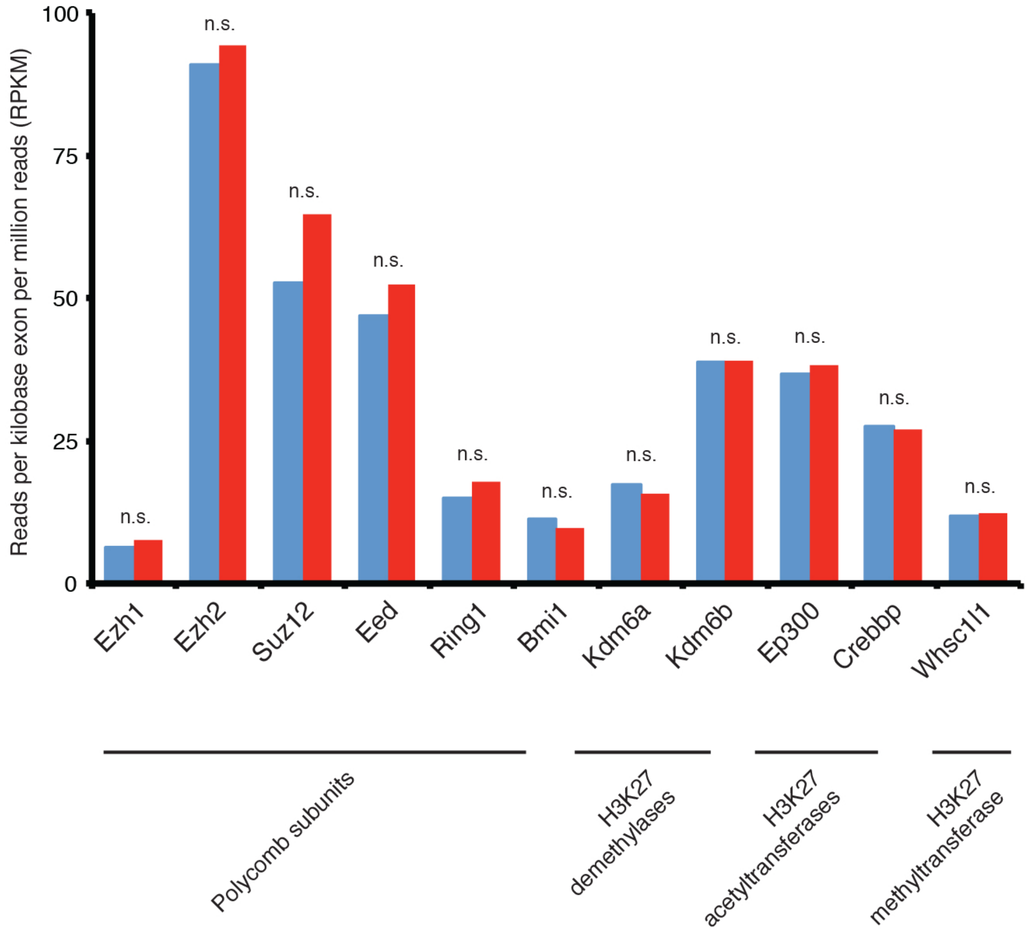
Reads per kilobase exon per million reads (RPKM)
100
75
50
25
0
n.s.
Ezh1
Ezh2
Suz12
Eed
Ring1
Bmi1
Kdm6a
Kdm6b
Ep300
Crebbp
Whsc1l1
Polycomb subunits
H3K27 demethylases
H3K27 acetyltransferases
H3K27 methyltransferase

Fig S8

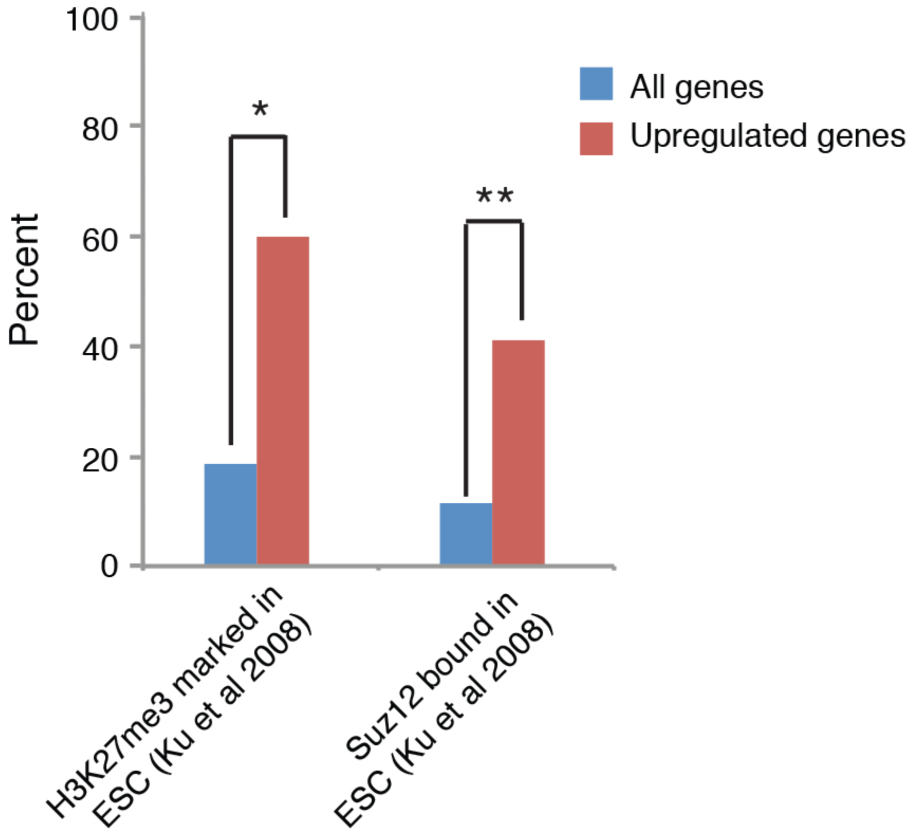

100
80
60
40
20
0
Percent
All genes
Upregulated genes
*
**
H3K27me3 marked in ESC (Ku et al 2008)
Suz12 bound in ESC (Ku et al 2008)

Supplementary Datasets

Table S1

Click here to Download Table S1

Table S2

Click here to Download Table S2

Table S3

Click here to Download Table S3

Table S4

Click here to Download Table S4
